# Surface Rolling Active Magnetic Emulsions

**DOI:** 10.1002/advs.202501866

**Published:** 2025-06-10

**Authors:** Muhammad Turab Ali Khan, Gaurav Gardi, Ugur Bozuyuk, Metin Sitti

**Affiliations:** ^1^ Physical Intelligence Department Max Planck Institute for Intelligent Systems 70569 Stuttgart Germany; ^2^ School of Medicine and College of Engineering Koç University Istanbul 34450 Turkey

**Keywords:** chemotaxis, self‐propelling droplets, surface rollers, micromachines

## Abstract

Active emulsions can exhibit chemotactic locomotion in fuel‐rich conditions like their biological counterparts. However, possessing extensive control over the spontaneous motion of chemotactic droplets and achieving their locomotion in fuel‐deficient environments remains as a challenge. Here, synthetic rotational flows are incorporated to augment droplets with on‐demand surface rolling ability. These rotational flows arise from a freely rotating magnetic cluster encapsulated within an oil droplet and aid in locomotion in fuel‐deficient regions. Combining autonomous and synthetic flows aids in switchable locomotion modes enabling active magnetic droplets to explore confined spaces by avoiding concentration gradient traps and entering narrow spaces. Finally, on‐demand switchability between surface rolling and autonomous swimming modes allows the active magnetic droplets to locomote against chemotactic gradients, while transporting and manipulating surrounding micro/nanoscale entities.

## Graphical abstract

This study presents synergistic combination of materials to augment the life‐like chemotactic abilities of oil droplets towards microrobotics applications. Magnetically actuated autonomous oil droplets possess on‐demand switchable locomotion modalities with augmented navigation in complex confined spaces and can perform cargo transportation.

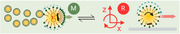

## 1 Introduction

Fluidic flows facilitate the transfer of mechanical torques,^[^ ^1^, ^2^ ^]^ information,^[^ ^3^, ^4^ ^]^ and materials^[^ ^5^ ^]^ across phase‐separated fluids. An evident example of such a mediation is demonstrated by the coupling of cytoplasmic shears across a cellular membrane for locomotion and sensing in microorganisms.^[^ ^6^, ^7^, ^8^ ^]^ Active emulsions (i.e., oil droplets in aqueous surfactant solutions) are utilized as an emerging model system for studying fluidic couplings across phase‐separated systems.^[^ ^9^, ^10^ ^]^ Interfacial activity of surfactants induces coupled fluidic flows both within and outside of the emulsified droplets.^[^ ^11^ ^]^ Such, emergent fluidic interactions render active emulsion life‐like chemotactic locomotion and sensing to be able to navigate through complex confined spaces.^[^ ^12^, ^13^ ^]^ However, these autonomous behaviors remain dependent on the availability of chemical fuels and directions of local chemical gradients, with material‐specific locomotion modalities. On the other hand, their biological counterparts can demonstrate adaptive locomotion modalities and can locomote in fuel‐deficient regions.^[^ ^14^, ^15^ ^]^ Thus, there is a dire need to positively augment the existing capabilities of active droplet systems for further utilization as effective model systems^[^ ^16^ ^]^ and in microrobotic applications.^[^ ^17^ ^]^

In the attempt to solubilize the active droplet, the interfacial activity of the surfactants induces surface tension gradients, resulting in fluidic flows along the gradients and causing the oil droplet to self‐propel (active droplet). These solubilization‐driven droplets self‐propel like pushing Squirmer^[^ ^18^, ^19^ ^]^ and the mode of locomotion is entangled with the molecular properties of the oil.^[^ ^9^, ^20^ ^]^ Furthermore, the direction of locomotion is guided by the local gradients in surfactant (fuel) concentrations.^[^ ^10^ ^]^ While in the absence of excessive surfactants, the droplets remain stationary as no or little solubilization occurs. Therefore, as a model system, droplet's behavior remains bound to pre‐determined environmental conditions and lack robotic functionalities. Overcoming these challenges necessitates a different driving mechanism to augment the capabilities of these active emulsions. Here, we propose the generation of out‐of‐plane rotational flows to render the soft spherical active oil droplets with a new locomotion modality, i.e., surface rolling on nearby boundaries. Surface rolling enables oil droplets to locomote in low‐surfactant regions, translate at higher propulsion speeds and even go against the chemotactic gradients. Eventually, switchable locomotion modes would act as escape maneuvers for autonomous swimmers in complex confined regions and aid in cargo transportation using active droplets.

## 2 Results

### 2.1 Rolling Dynamics of Passive Magnetic Emulsions

**Figure 1:**
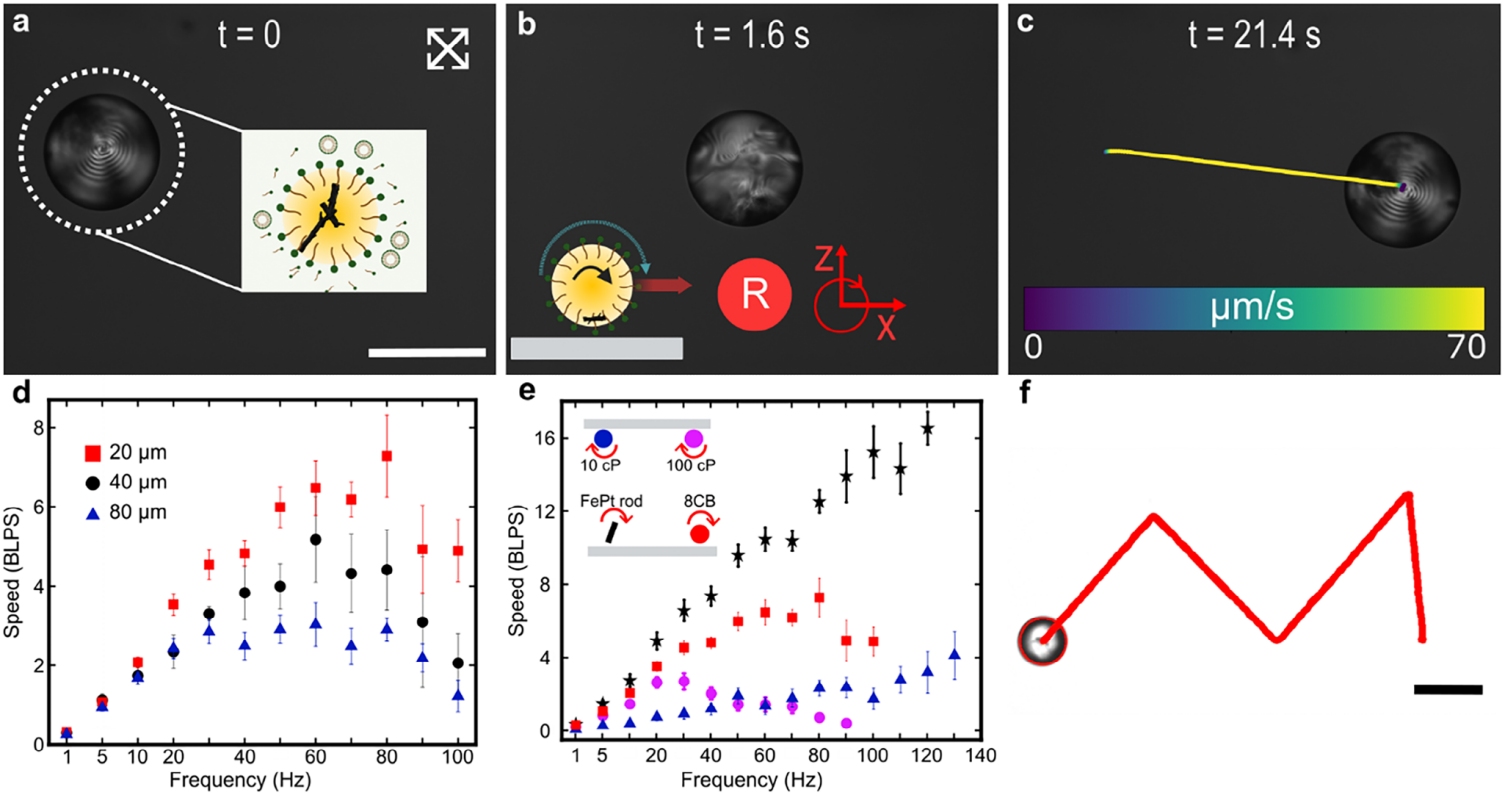
Surface rolling behavior of passive magnetic droplets encapsulating a cluster of ferromagnetic FePt nanoparticles. a–c) Time series for evolution of polarized optical microscopy (POM) texture of a droplet under 5 mT, 10 Hz out‐of‐plane rotating magnetic field at low micellar concentrations (passive state). a) Equilibrium ordering (t = 0 s), b) disrupted POM texture while magnetic cluster is rotating (t = 1.6 s), and c) restored POM texture after removal of applied field (t = 21.4 s). d) Translation speed of rolling droplets with respect to droplet diameter at frequencies ranging from 1 to 100 Hz and 10 mT magnetic field in a chamber with 2 mm height. e) Comparison of the average translation speed of 8CB, 10 cP, 100 cP silicone oil droplets and FePt rods (≈20 µm in diameter) as a function of magnetic field frequency at 10 mT in a chamber with height 2 mm. f) Precise locomotion of surface rolling droplet on an ‘M‐shaped’ trajectory. Error bars in (d,e) indicate 95% confidence interval for a population of respective droplets with varying cluster sizes. Scale bar is 100 µm in (a,f).

We study the influence of rotational flows generated by a rotating solid magnetic cluster encapsulated inside an emulsified oil droplet, under the application of external magnetic fields. The encapsulated magnetic cluster is composed of many ferromagnetic FePt nanoparticles and is free to rotate within the emulsified oil droplets. These magnetic droplets are stabilized by interfacially adsorbed surfactant (TTAB, Tetradecyltrimethylammonium bromide) molecules from the surrounding aqueous media. To purely consider the influence of the cluster rotation on the droplet, we begin with passive/stationary droplets at 1 wt.% surfactant (TTAB) concentrations where no substantial solubilization related fluidic flows arise. We verify this by choosing nematic oil (8CB, Octyl‐4‐biphenylcarbonitrile) and visualize the internal flows using polarized optical microscopy (POM). Initially, the nematic droplet remains static with equilibrium POM texture (+1 radial hedgehog defect) arising from absorbed TTAB molecules at the interface (**Figure** **1**a).^[^ ^21^ ^]^ Upon the application of rotating external magnetic fields, the magnetic cluster synchronously rotates inside the droplets and generates rotational flows. These flows distort the equilibrium POM texture (Figure 1b; Movie S1, Supporting Information) and influence the oil/water interface to rotate. The translation of flows to the surrounding aqueous media and the presence of a nearby boundary lead to symmetry breaking in the flow profile, influencing the droplets to roll on the substrate (nearby boundary) and translate along the plane of rotation. Eventually, as we cease the cluster rotation, the droplet and the rotational flows come to a halt, resulting in restoration of the equilibrium POM ordering (Figure 1c).

The rolling dynamics of the magnetic droplets on nearby boundaries can be influenced by various factors. We begin with investigating the effect of the rotational frequency of the external magnetic field on droplets sized ≈80 µm (Figure 1d, blue triangles; Movie S2, Supporting Information). We observe that the translation speeds of ≈80 µm‐diameter droplets increase linearly with increments in the frequency till 30 Hz. Beyond 30 Hz, the translation speeds plateau and further increments in the frequency of the rotating magnetic field do not significantly enhance the translation speeds. Such saturation is not expected as the rotational flows and the resultant translation speeds of surface rollers increases linearly with the frequency of rotating external magnetic fields. In rolling droplets, the strength of the rotational flows and the observable translation speeds are governed by the rotational dynamics of the encapsulated magnetic cluster. To understand the saturation of the speeds, we investigate the motion and behavior of the magnetic cluster inside the emulsion at different frequencies (Movie S3 and Supplementary text). We find that at lower frequencies, the cluster rotates as a single body and undergoes 3D motion inside spherical emulsions. The motion of the magnetic cluster is quantified by tracking its 3D movement inside the spherical confinement by viewing the droplets from top (x‐y plane) and side (z‐x plane), while they are rolling (Figures S1–S4, Supporting Information). It can be observed that rotational motion of the cluster generates rotational flows that cause the droplets to roll on the substrate (Movie S3, Supporting Information). At higher rotational frequencies (beyond 10 Hz), the cluster breaks into smaller rotating clusters and the extent of breaking depends on the frequency of the rotating magnetic field (Figures S5 and S6, Supporting Information). This breaking is expected as the encapsulated magnetic cluster is an aggregate of the many FePt nanoparticles held together by magnetic dipole‐dipole interactions. These interactions are overcome by fast rotating fields and smaller rotating clusters are observed.^[^ ^3^, ^4^ ^]^ The reduction in the size of the rotating magnetic clusters reduces the strength and the length scale of the generated rotational flows (Figure S7, see Supporting Information for more details). However, increments in the frequencies causes the clusters to rotate faster, leading to competing effects on the net generated rotational flows and the plateauing of the translation speeds beyond 30 Hz.

Since surface rolling dynamics of the droplets are influenced by the length scale of the rotational flows, we compare the translation dynamics of differently sized droplets (Figure 1d). We find that smaller droplets translate faster in terms of body lengths per second (BLPS) at all applied frequencies and the saturation of translation speeds is observed at 60 Hz. This is expected since the distance from the center of the cluster to the substrate reduces and the rotational flows are more efficiently translated from the cluster's surface (Figure S8, Supporting Information). We also observe that the translation speeds of all sizes start to fall at and beyond 90 Hz, indicating that the magnetic cluster is not rotating synchronously with the applied magnetic field (Movie S2, Supporting Information). As different‐sized 8CB droplets step out at the same frequency, we suspect the influence of 8CB's internal viscosity (≈35 cP) on the cluster rotation dynamics.^[^ ^22^, ^23^ ^]^ To investigate further, we measure the translational speeds of the FePt rods (length ≈20 µm) in 1 wt.% TTAB solution (Figure 1e). We find that the rolling speeds of the magnetic cluster continue to increase till 130 Hz and until then it does not step‐out. This indicates that stepping out of the nematic emulsions is related to the internal viscosity of the oil. To further test this hypothesis, we experiment with two isotropic silicone oil droplets of different viscosities (≈10 and ≈100 cP) with an encapsulated FePt magnetic cluster. Upon comparing the translation speeds of the same size group (≈20 µm), we find 100 cP silicone oil droplets step‐out beyond 30 Hz while 10 cP droplets continue to roll at increasing speeds till 140 Hz. Thus, the internal viscosity of the oil plays a critical role in the rotational dynamics of the encapsulated magnetic cluster and resultantly influence the rolling dynamics of the droplets. Finally, we demonstrate effective control over the droplet translation by controlling the external magnetic field's plane of rotation (Figure 1f), and patterning the letter ‘M’.

### 2.2 Hydrodynamic Mechanism of Surface Rolling Droplets

We have experimentally investigated various parameters that have an impact on the rolling dynamics of the droplets. Now we begin to unravel the underlying hydrodynamic mechanisms for surface rolling droplets. The rotational and translational motion of the magnetic cluster leads to generation of internal flows. In general, internal flows from the motion of encapsulated particles can cause the droplets to both translate and rotate.^[^ ^24^, ^25^, ^26^, ^27^, ^28^ ^]^ In our system, oil droplets are located close to the substrate, therefore, the motion of the droplets is also affected by the lubrication distance between the oil/water interface and the substrate.^[^ ^29^, ^30^, ^31^, ^32^ ^]^ Another factor to consider is droplet's deformation, the experimentally studied systems did not demonstrate any noticeable deformation. This is expected as the surface tension effects dominate both gravitational and shear forces (Supplementary text). The spherical shape brings locomotion dynamics of droplets closer to the solid‐body locomotion, however, the differences arise due to viscous dissipation from internal circulation. Internal circulation in droplets is influenced by the ratio of droplet's dynamic viscosity (µ_*d*_) to external fluid's dynamic viscosity (µ_*ext*_).^[^ ^28^, ^33^ ^]^

(1) $$\lambda =\frac{\mu_{d}}{\mu_{ext}}$$

The viscosity ratio (λ) plays a critical role in determining the stokes drag acting on the translating droplet.^[^ ^28^ ^]^ The drag acting on the droplet gets further modified if an encapsulated particle, located at the center of the droplet, is draged by external force.^[^ ^27^ ^]^ Finally, wall correcting factors need to be introduced to compute drag on the droplet driven by the translational motion of an encaspulated particle.^[^ ^30^, ^31^, ^32^, ^34^ ^]^

(2) $$F_{d}=12\pi \mu_{ext}RV\;\frac{2\varepsilon^{5}\left(\lambda -1\right)+3\lambda +2}{5\varepsilon^{2}-4\varepsilon^{5}\left(\lambda -1\right)-3\left(2\lambda +3\right)}\;C_{1}\left(\delta^{\prime }\right)$$

*R* is the radius of the droplet, *V* is the velocity of the droplet, ε is the radius of the encapsulated particle and *C*_1_(δ′) is the function of wall correcting factor. Furthermore, the viscosity ratio (λ) also determines the effective transfer of torque from a rotating encapsulated particle (Ω_*particle*_) to the droplet's interface (Ω_*d*_).^[^ ^33^ ^]^

(3) $$\Omega_{d}=\frac{1}{1+\lambda^{-1}\left(\alpha^{3}-1\right)\;}\;\Omega_{particle}$$

**Figure 2:**
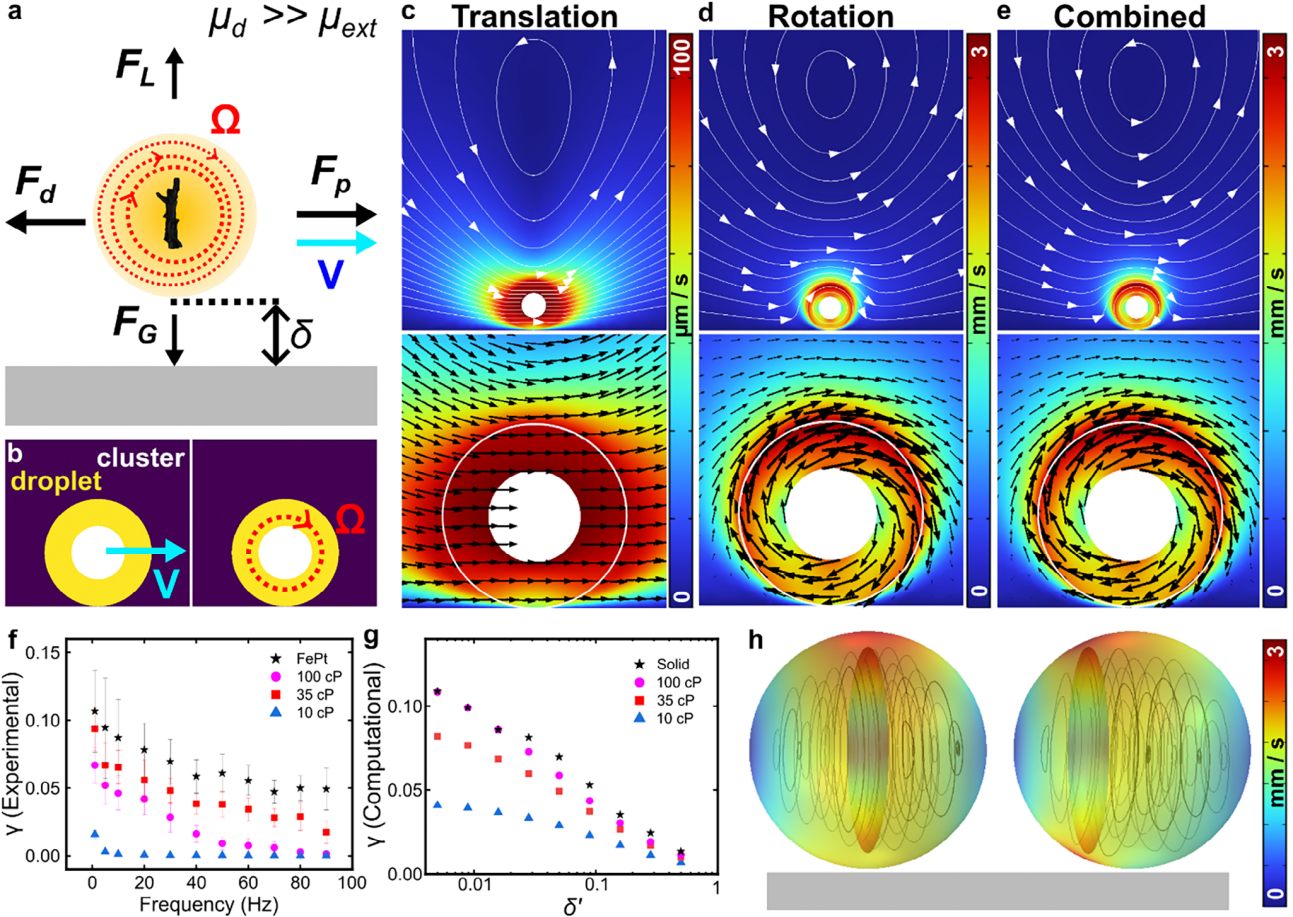
Computational fluid dynamic (CFD) simulations of surface rolling droplets. (a) Force balance on a surface rolling droplet. F_p_ represents the forward propulsion force balanced out by the drag force (F_d_) acting on the droplet. Resultantly, the droplet rotates with Ω and locomotes with a velocity V at a lubrication distance δ. δ is maintained by the balance between gravitational force (F_G_) and lift force (F_L_). (b) Explanation of the simulation: droplet is as defined as a sphere with sharp viscosity contrast across the interface. The magnetic cluster is modeled as a sphere translating with a prescribed velocity (V) or rotating with a prescribed omega (Ω) at the center of the droplet. (c‐e) External (top, streamlines) and internal (bottom, arrow surface) as a result of prescribing computationally predicted translational velocity (c), prescribing omega (d) to the encapsulated magnetic cluster, and combination of translational and rotational components of the flows from the encapsulated magnetic cluster (e). The dynamic viscosity of the simulated droplet (diameter 80 µm) is 35 cP and the cluster (diameter 40 µm) is rotated at 10 Hz. The outer white circle denotes the droplet's interface. The color bar represents the magnitude of velocity. (f) Experimentally determined effective slipping coefficient γ  =  *V*/*R*2π*f* as a function of the external magnetic field's frequency. (g) Computationally determined γ as a function of δ′ =  δ/*R*. (h) The velocity distribution and internal streamlines with respect to the position of the magnetic cluster. The color bar represents the magnitude of velocity. The external viscosity is 1 cP in all simulations.

α  =  *R*/ε is the ratio between the droplet's radius (*R*) and the encapsulated particle's radius (ε). Resultantly, the rotational drag acting on the droplet near a wall gets modified as follows,

(4) $$F_{p}=\pi \mu_{ext}R^{2}\Omega_{d}C_{2}\left(\lambda ,\varepsilon ,\delta^{\prime }\right)$$

where *C*_2_(λ,ε,  δ′) in F_p_ is the combined effect of viscosity contrast, the radius of the encapsulated particle, and the wall correcting factors. Since the droplet should not experience any net torque or force at low Reynold's number, we can now determine the parameters that influence the velocity of the droplets (**Figure** **2**a).

(5) $$V=\frac{R\Omega_{d}C_{2}\left(\lambda ,\varepsilon ,\delta^{\prime }\right)}{12\;C_{1}\left(\delta^{\prime }\right)}\;\frac{5\varepsilon^{2}-4\varepsilon^{5}\left(\lambda -1\right)-3\left(2\lambda +3\right)}{2\varepsilon^{5}\left(\lambda -1\right)+3\lambda +2}$$

It reveals that the locomotion velocity of the droplets is affected by the viscosity contrast across the interface (λ), lubrication factor (δ′ =  δ/*R*), the radius of the droplet (*R*) and the encapsulated particle (ε), and the angular velocity of the droplet (Ω_*d*_). The viscosity ratio (λ) and the droplet's radius (*R*) are predetermined and remain constant for the chosen oil/water system. The angular velocity of the droplet is a function of the rotational frequency of the cluster, alongside, the cluster's size and position within the droplet (Equation 3).^[^ ^28^, ^33^ ^]^ The rotational frequency of the cluster can be controlled externally. However, the position of the cluster evolves over time (Figure S3 and Movie S3, Supporting Information) and size varies with respect to applied rotational frequency (Figure S5, Supporting Information). Finally, the lubrication factor (δ′) is a balance between gravitational (F_G_) and the lift force (F_L_) due to repulsion from the substrate (Figure 2a). δ′ cannot be controlled externally, however, it has been previously reported to be increasing as a function of the angular frequency and velocity of the surface roller.^[^ ^34^, ^35^ ^]^

We carry out CFD simulations to understand the influence of these factors and compare the findings with experiments. In simulations, we define a spherical droplet with a circular rotating magnetic cluster (Figure 2b). The oil/water interface is defined by a sharp viscosity contrast. We begin with fixing the position of the spherical magnetic cluster at the center of the droplet. By doing this, the translational motion of the cluster parallel to the substrate is not expected to render rotational velocity to the droplet.^[^ ^28^ ^]^ Simultaneously, a rotating spherical cluster at the center of the droplet is not expected to translate the droplet,^[^ ^28^ ^]^ unless the symmetry is broken by the substrate. The forces acting on the droplet due to the translation or rotation of the encapsulated particle are evaluated by computing the difference in traction on the interior (σ_*int*_) and exterior (σ_*ext*_) of the droplet's interface.^[^ ^32^ ^]^

(6) $$F=\int \;\;\;\int \left(\sigma_{ext}-\;\sigma_{int}\right)\cdot ndS$$

n is the normal vector pointing outwards from the interface, and S is the droplet's interface. Using the traction forces and following the validation exercise for the simulations,^[^ ^29^, ^32^, ^34^, ^36^ ^]^ we determine the computational velocity of the droplet resulting from the translational and rotational motion of the encapsulated cluster (Figure S9, Supporting Information). We then begin to unveil the dominant factor between rotational and translational flows from the motion of the encapsulated cluster. Figure 2c shows the effect of prescribed velocity to the encapsulated cluster and the resulting flow fields inside and outside the droplet. The internal and external flow fields differ for the prescribed rotation to the encapsulated cluster (Figure 2d). The summation of the two effects qualitatively indicates that the rotational flow dominantly contributes to the locomotion of the droplet on the substrate (Figure 2e).

We find quantitative agreement in the experimental and computational effective slipping coefficient.

(7) $$\gamma =\frac{V}{R2\pi f}\;$$

f represents the frequency of the applied external rotating magnetic field. We utilize the applied rotational frequency because of the lack of access to both the computational and experimental rotational frequency of the droplet. The effective slipping coefficient (γ) is a quantitative measure for the rolling efficiency of the surface roller.^[^ ^34^ ^]^ Experimentally γ decays as function of the magnetic field's frequency (Figure 2f). This can be expected as the increase in the rotational frequency and speed of the droplet leads to higher lift force and increases the δ′.^[^ ^35^ ^]^ The simulations also demonstrate a decreasing γ as δ′ increases (Figure 2g). Furthermore, the viscosity contrast palys a critical role as highly viscous droplets behave similarly to solid surface rollers at very small δ′.^[^ ^32^ ^]^ The simulations and the experiments demonstrate an acceptable agreement for solid rollers and 35 cP droplet (8CB) rollers. However, the experimental γ for 100 cP and 10 cP droplets is not in agreement with the simulations. The silicone oil droplets have lower density and roll on the top substrate and in this case, gravity adds up with the lift and repulsion force, balanced by the buoyancy. Therefore, deviations are expected between simulations and experiments as our simulation only investigates the effect of viscosity contrast on rolling dynamics and does not include buoyancy. Nonetheless, the trend demonstrated by the simulation for droplets with different viscosities is consistent with the previously reported results.^[^ ^32^ ^]^ Furthermore, we qualitatively compare the velocity distribution over the surface of the droplet and the resulting internal flows with respect to the position of the rotating cluster (Figure 2h). The redistribution of velocity as a function of the cluster's position further distinguishes a droplet roller from a solid surface roller. The position of the cluster within a droplet evolves due to interactions with the internal flows.^[^ ^37^ ^]^ These interactions become more complex as the cluster breaks into many small rotating clusters (Movie S3, Supporting Information). The resultant internal flow structures and the tractions experienced by the many small rotating clusters inside spherical droplet demand further investigation. However, we limit the scope of this work to the net influence of cluster rotation and transfer of torque to the droplet's interface (Equation 3) that governs the rolling dynamics of the droplets.

### 2.3 Rolling Dynamics of Active Magnetic Emulsions

**Figure 3:**
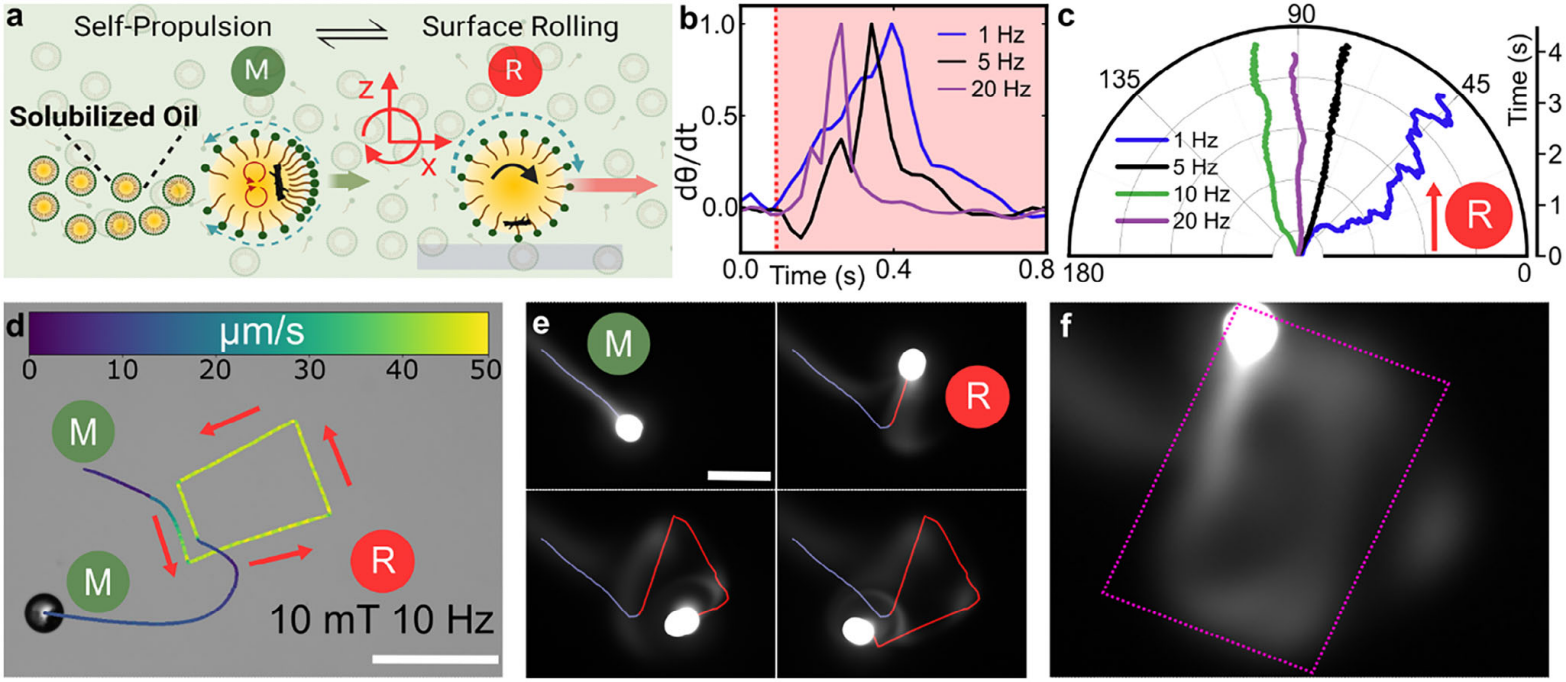
Reversible switching between translational self‐propulsion and magnetic rotation‐based surface rolling locomotion modes of an active magnetic droplet. a) A self‐propelling droplet (M) via Marangoni flows at the interface (blue) and inside (red) switches between self‐propulsion and surface rolling modes using out‐of‐plane rotating fields. The droplets leave behind a trail of solubilized oil (filled micelles). b) Time required for the change in a droplet's trajectory at 1, 5, and 20 Hz interpreted in terms of normalized change in the angle of propulsion with respect to the *x*‐axis (dθ/dt, where θ represents the angle of propulsion of the droplet). (c) Droplet trajectory versus time after the application of out‐of‐plane rotating magnetic fields at 1, 5, 10, and 20 Hz. The plane of rotation is at 90° with a magnetic field strength of 10 mT. d) Switching between self‐propelling and surface rolling modes using out‐of‐plane rotating fields (160‐µm chamber height). e) Image sequence of the evolution of chemical trail using Nile red‐doped self‐propelling droplet visualized under fluorescence microscopy. f) Evolved square pattern of the chemical trail. Color bar in (d) represents the instantaneous speed in µm s^−1^. The scale bar is 200 µm in (d,e).

In low surfactant concentrations and in the absence of a magnetic field, the droplets and the magnetic cluster remain stationary as there are no rotational or solubilization‐related fluidic flows to propel them. Upon increasing the surfactant concentration, the droplets begin to self‐propel due to fluidic flows (Marangoni flows).^[^ ^11^ ^]^ These flows originate from the spontaneous symmetry breaking of the surfactant distribution on the oil‐water interface by excessive surfactants. Concurrently, these spontaneous flows dynamically orient the encapsulated magnetic cluster perpendicular to the propulsion direction (Figure S10, Supporting Information).^[^ ^38^ ^]^ To observe the rolling dynamics of droplets on nearby boundaries in the presence of Marangoni flows, we confine self‐propelling droplets in a 160 µm‐height glass cell to force the droplets to roll on either of two glass boundaries. Such confinement can slightly reduce the rolling speeds of the droplets in comparison to vertically unconfined environments (Figure S11, Supporting Information).^[^ ^34^ ^]^ As the droplet self‐propels at 20 wt.% TTAB (solubilization rate ≈0.17 µm min^−1^ ± 0.06 µm min^−1^), we begin to observe the rolling dynamics in the presence of the Marangoni flows. We find that the application of the magnetic field causes the magnetic cluster to rotate even in the presence of Marangoni flows and enables the droplet to switch to surface rolling (**Figure** **3**a). However, the switching dynamics are governed by the frequency of the applied magnetic field. It takes a shorter time for the droplet to switch to a directional surface rolling as the magnetic field's frequency increases (Figure 3b). Consecutively, the final direction of propulsion via surface rolling is also affected by the frequency of the applied magnetic field (Figure 3c). A magnetic field rotating at 1 Hz and with a plane of rotation at 90° would drive the droplet at ≈45° with periodic changes in the propulsion direction (Figure S11, Supporting Information). While the same droplet propels smoothly along the plane of rotation when the magnetic field rotates at 20 Hz. Thus, switching to efficient surface rolling necessitates strong rotational flows to overwhelm the spontaneously occurring Marangoni flows.

Using higher magnetic field frequencies, the switch to surface rolling can be precisely controlled by changing the magnetic field plane of rotation (Figure 3d; Movie S4, Supporting Information). Simultaneously, the droplet's propulsion speed is higher in the surface rolling mode. Upon switching off the magnetic field, the droplet switches back to self‐propulsion and continues to swim autonomously. Additionally, we study the switching dynamics to surface rolling for other isotropic emulsions that are initially propelling with Marangoni flows and we find that droplet propulsion dynamics can be effectively manipulated by stronger out‐of‐plane rotational flows, irrespective of the chemical composition of the oil (Figure S13, Supporting Information). Furthermore, we investigate the influence of these rotational flows on the droplet trails. Solubilizing droplets leave a trail of dissolved oil, which plays an essential role in mediating interactions between droplets^[^ ^39^, ^40^ ^]^ and in some cases hinders the control over propulsion directions.^[^ ^38^ ^]^ A self‐propelling droplet performs a straight‐line deposition of the dissolved oil trail along its trajectory (Figure 3e). However, switching to surface rolling allows the droplet to manipulate its own trail without being affected by it. Finally, rolling in a square trajectory leads to the evolution of a square pattern of the solubilized oil trails (Figure 3f; Movie S4, Supporting Information). We do not observe any significant reduction in the size of the droplets upon exposure to out‐of‐plane rotating magnetic fields (for short times <2 min). The solubilization dynamics during rolling at higher frequencies (for longer times, ≈hours) remains as a subject of future exploration. Furthermore, the droplets’ shape and interfacial dynamics remain preserved as the emulsions reversibly switch to self‐propulsion, evidencing the minimally invasive nature of this control method. Additionally, the ability to manipulate droplet trails opens exciting opportunities for manipulating neighboring micro/nanoobjects and influencing the collective behaviors of interacting droplets.^[^ ^18^, ^41^ ^]^

### 2.4 Augmented Navigation in Confined Spaces

**Figure 4:**
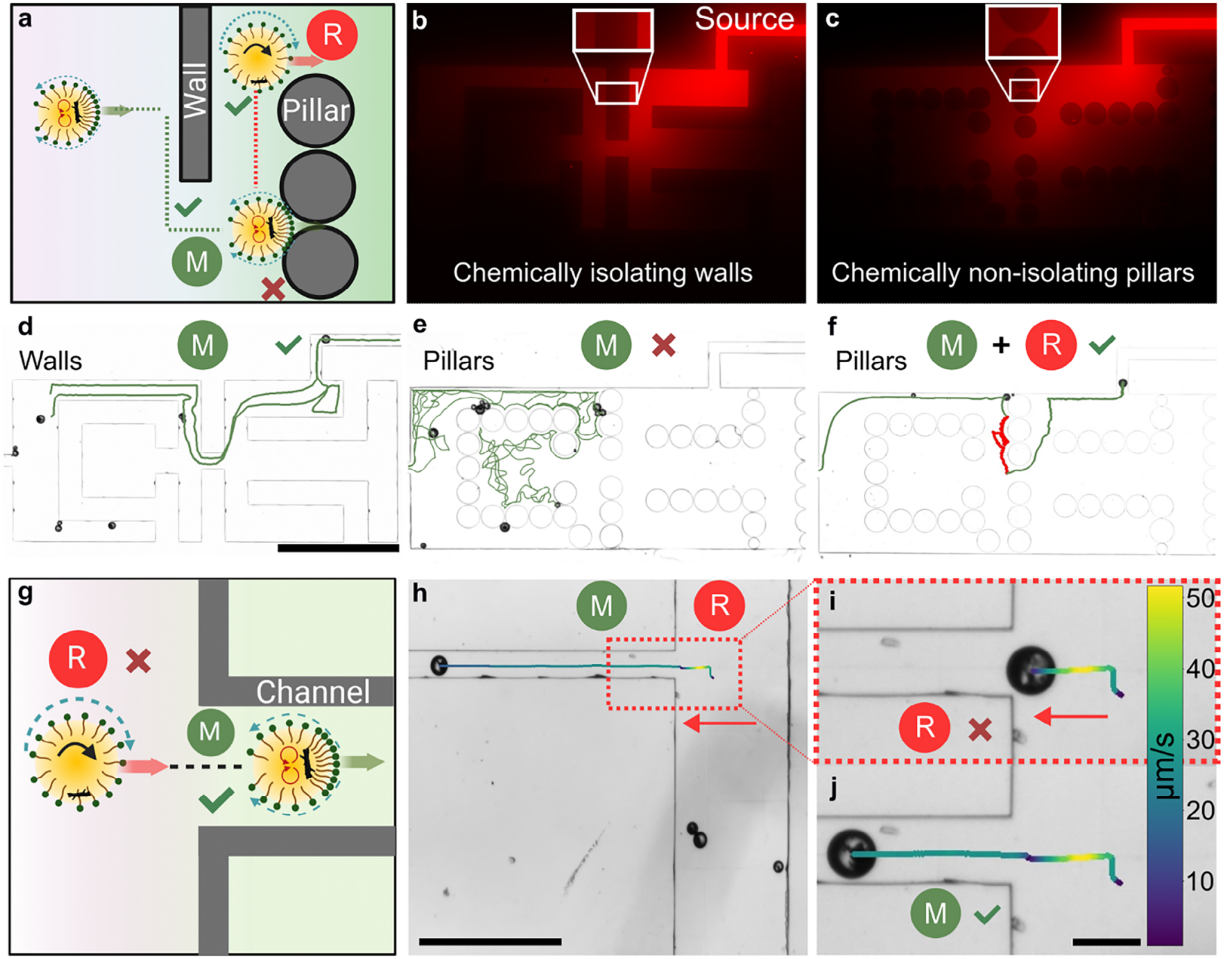
Switchable two locomotion modes of active magnetic droplets augment chemotactic navigation in confined spaces. a) Schematic depicting the motion of a self‐propelling droplet along a chemical gradient and interaction with obstacles on its way. Visualization of chemical gradients using fluorescence microscopy in b) chemically isolating walls and c) diffusion of surfactants through pillars in chemically non‐isolating pillars. d) Trajectory of two leading droplets that successfully navigate through a chemically isolating environment. e) Trajectory of four leading droplets navigating in a non‐isolating environment, where all droplets get stuck at pillars and fail to navigate through the region. f) Trajectory of a droplet successfully navigating through the chemically non‐isolating environments by switching between locomotion modes. g) Schematic depicting switchable locomotion enabling entry into narrow channels. h–i) Droplet accelerating towards the channel via surface rolling. Entry into the channel via surface rolling is prohibited by the flow field around the rolling droplet. j) Upon switching off the magnetic field, the droplet enters the channel due to propulsion driven by Marangoni stresses. Trajectories in (h‐j) are color coded in instantaneous speed, with color representing speeds in µm s^−1^. Scale bar represents 1 mm in (d), 500 µm in (h), and 100 µm in (i,j).

Richness in the locomotion modes and on‐demand switching between modes of locomotion allow us to augment life‐like chemotactic abilities of oil droplets to navigate through confined spaces. The chemotactic nature of these droplets arises from the advection towards higher empty micelle concentrations and this ability is useful for navigation through complex environments.^[^ ^13^ ^]^ We study how a pre‐established chemical gradient guides self‐propelling droplets to navigate through chemically isolating and non‐isolating regions (**Figure** **4**). Figure 4b shows a visualization of a patterned chemical gradient in a confined space with isolating walls that only allow for the adsorption of surfactant molecules but not diffusion through them. Under these conditions, self‐propelling droplets containing a magnetic cluster navigate their way toward higher micelle concentration and the trajectory of the leading droplets is the shortest path out of the region (Figure 4d; Movie S5, Supporting Information). Complexities arise for the droplets following them as they encounter repulsive‐filled micelles generated by the leading droplets (Figure S14, Supporting Information).

Because the composition of confined spaces is not limited to chemically isolating walls, purposeful chemotactic navigation should allow for adaptive navigation through chemically non‐isolating regions (Figure 4a). To test the chemotactic capability of self‐propelling droplets (20–80 µm in diameter) in navigating through a chemically non‐isolating confined space, we replace walls with 10 µm apart micropillars (100‐µm height and 200‐µm diameter) without changing the rest geometry of the environment. Visualization of the patterned chemical gradient indicates that spaces between the pillars allow for diffusion of surfactant molecules through them, creating local concentration gradient traps (Figure 4c). Chemotactic navigation guides the leading droplets to the pillars nearest to the inlet of free micelles (Figure 4e). Droplets remain stuck in between these pillars, occasionally moving to the adjacent pillar until they are completely solubilized and only the magnetic cluster remains (Figure S15, Supporting Information). Pre‐patterned chemical gradients offer small supplies of the chemical food through narrow spaces that strongly inhibit the droplet's ability to navigate through chemically non‐isolating environments.

Therefore, we utilize external magnetic control to avoid these concentration gradient traps and aid in navigation through such tight regions. We switch to surface rolling to extract trapped droplets out of the pillars and switch back to Marangoni stress‐driven propulsion at wider openings, where a droplet finds its path toward a higher concentration gradient and exits the region (Figure 4f). Extraction of a single droplet is possible via surface rolling; however, the global magnetic control only allows for focusing on a single droplet at once. Therefore, the trailing droplets that also get stuck at the pillars are extracted individually (Figure S16a–c, Supporting Information). We observe that two out of three droplets find their way out of the trap (Figure S16d,e, Supporting Information). The increasing flux of the repulsive filled micelles near the pillars repels the droplets, causing the last droplet to find its way out of the region by dint of auto‐chemotactic interactions (Figure S16f, Supporting Information).^[^ ^13^ ^]^

In the absence of a global chemical gradient, the leading self‐propelling droplet can exit through the region after some exploration, due to Marangoni‐assisted propulsion (Figure S17, Supporting Information). However, the trailing droplets experience strong negative autochemotaxis and cannot exit the region. Combining Marangoni‐assisted propulsion with surface rolling aids in navigation through such regions (Figure S17, Supporting Information). On this note, it is also essential to understand which locomotion mode is suitable for entering the narrow channels. We find that a self‐propelling droplet can seamlessly enter narrow spaces (Figure 4g; Movie S6, Supporting Information). However, a surface rolling droplet can get stuck near the entrance of the channels and rotate there without translating into the channel (Figure 4h,i).^[^ ^34^ ^]^ The hydrodynamic flow profile around a surface rolling droplet causes a repulsive force in front of the channel entrance and prevents the droplet from entering the channel. This behavior is expected to depend on the droplet size (the ratio between droplet and channel diameter) as the hydrodynamic flows depend on the size and distance from the droplet. However, switching off the rolling motion allows the droplet to switch to self‐propulsion with a weaker hydrodynamic flow profile around it.^[^ ^38^ ^]^ Therefore, the droplet seamlessly enters the narrow channel (Figure 4j). Thus, surface rolling helps to navigate regions with repulsive filled micelles, while Marangoni‐assisted propulsion enables entry into narrow spaces that rolling droplets cannot enter due to the hydrodynamic flow fields around them (Figure S18, Supporting Information).

### 2.5 Neighboring Microparticle Swarm Transport

**Figure 5:**
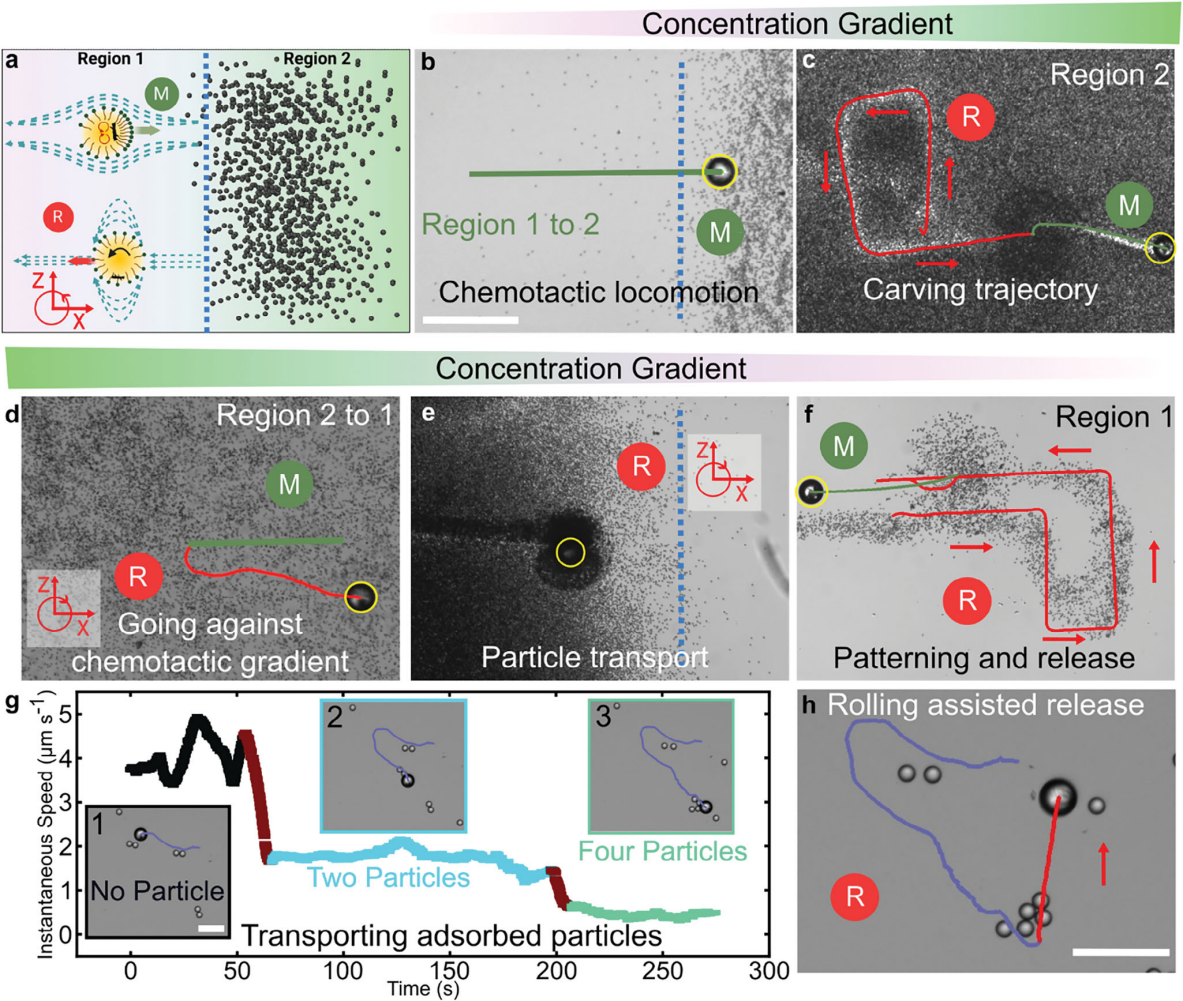
Neighboring microparticle swarm transportation, release, and patterning using multiple locomotion modes of the proposed self‐propelling droplets. a) Schematic illustration of the experimental conditions for b–f), where the blue line distinguishes Region 1 ([TTAB] = 1 wt.%) and Region 2 ([TTAB] = 20 wt.%) and is visualized by 3 µm‐diameter polystyrene microparticles dispersed in 20 wt.% TTAB. b) Chemotactic locomotion from Region 1 to 2. c) Carving precise trajectory by removing particles along the path of rolling droplet. d) Moving against chemical gradients using surface rolling. e) Transporting trapped particles against chemotactic gradients to Region 1. f) Precise patterning of trapped particles by changing the plane of rotating magnetic field and spontaneous release of trapped particles by switching to Marangoni‐assisted self‐propulsion. g,h) Transporting 20 µm‐diameter mesoporous silica particles. Silica particles’ surfaces are silanized to enable adsorption on the surface of the droplet. g) Instantaneous speed of a magnetically steered self‐propelling droplet (50 µm‐diameter) with no particle adsorbed on its surface (1), two particles adsorbed at its surface (2), and four particles adsorbed on its surface (3). h) Rolling assisted release of adsorbed particles under out‐of‐plane rotating magnetic fields. Red arrows in (c,f) indicate the droplet's rolling direction. The yellow circle in b‐f is added to highlight the droplet. Scale bar is 200 µm in (b,h) and 50 µm in (g).

The hydrodynamic flow profile around the droplet varies with respect to the locomotion mode and these fluidic flows cause the droplet to interact with the surrounding passive particles (**Figure** **5**a). We find that switching between these flow profiles aids in cargo seeking, transportation, patterning, and release. We design an experimental chamber with two distinct regions; Region 1 with 1 wt. % TTAB and Region 2 containing 3 µm‐diameter polystyrene particles dispersed in 20 wt. % TTAB. The concentration gradient from Region 2 to 1 attracts the droplets, resulting in chemotactic advection towards Region 2 (Figure 5b). In Region 2, we switch to surface rolling to trap cargo particles within the flow fields around the droplet^[^ ^36^ ^]^ and carve trajectories in densely packed environments (Figure 5c; Movie S7, Supporting Information). Switching back to self‐propulsion results in the release of the trapped particles as the hydrodynamic flow field around a self‐propelling droplet is weak.^[^ ^38^ ^]^ Surface rolling also allows droplets to locomote against concentration gradients and transport trapped particles along with them (Figure 5d). During transportation, we observe that the trajectory is marked by residual particles, and we pattern them as ‘P’ by precisely controlling the direction of surface rolling (Figure 5f). Finally, switching to self‐propulsion releases the trapped particles and the droplet goes towards the concentration gradient and inevitably seeks more particles. Furthermore, the droplet's liquid nature also allows for studying particle transport via chemical interactions. We design a cargo by modifying the surface of silica particles (see methods) to enable the adsorption of this cargo on the oil‐water interface. We demonstrate the seeking, manipulation, and transportation of these interfacially adsorbed particles by magnetically steering the propulsion direction using static magnetic fields.^[^ ^38^ ^]^ We note that the instantaneous speeds of the self‐propelling droplet reduce as the number of adsorbed particles increases (Figure 5g). Finally, we release these particles by switching to rolling using out‐of‐plane rotating magnetic fields (Movie S8, Supporting Information).

## 3 Discussion and Conclusion

We proposed the utilization of synthetically generated rotational flows to transport passive oil droplets and augment the locomotion modalities of self‐propelling active droplets. These rotational flows enable the droplets to roll on near‐by boundaries and offer extensive control over the propulsion dynamics of self‐propelling droplets. Magnetic actuation‐based surface rolling enables on‐demand overwhelming of Marangoni flows and allows translation at increased speeds and even movement against chemical gradients. We demonstrated the utilization of switchable locomotion modes for entering and navigating through confined spaces. Finally, the combination of the two locomotion modes allows for neighboring microparticle swarm transporting, patterning, and release. Thus, a combination of synthetic rotational flows and autonomous Marangoni flows opens room for potential applications; however, several challenges need to be addressed.

The rolling ability of an active magnetic droplet is only tested in a Newtonian fluid at relatively lower viscosities. The rolling dynamics of droplets need to be investigated for propulsion in both viscous and non‐Newtonian fluids.^[^ ^42^, ^43^ ^]^ Along similar lines, a combination of the rolling and self‐propulsion have been studied at lower Peclet numbers, where the Marangoni flow profile remains steady.^[^ ^18^ ^]^ It remains an open question to either control or overwhelm the Marangoni flows at higher Peclet numbers where these solubilization flows become chaotic.^[^ ^44^ ^]^ Furthermore, the collective dynamics of the many rotating magnetic clusters and the resulting internal flow structures remain a subject of future exploration. Finally, successful navigation through switchable locomotion modes remains limited to one droplet at a time due to globally uniform magnetic fields. Utilization of the non‐uniform magnetic fields may allow for the extraction of many droplets simultaneously.^[^ ^45^, ^46^ ^]^

Controllable chemotactic droplets can be useful for in situ biosensing applications,^[^ ^47^ ^]^ such as observing localized environmental cues. For example, surface rolling can be used to explore wide regions while the POM texture provides optical feedback of the events happening in the vicinity or inside the liquid crystalline droplets. Combining autonomous and externally induced flows may also augment the abilities of droplets to navigate upstream against external flows^[^ ^48^, ^49^ ^]^ and may be useful in existing emulsion‐based drug‐delivery applications.

## 4 Experimental Section

### Materials

The following chemicals were used as received without any further purification or modification: 4‐octyl‐4'‐cyanobiphenyl (8CB, Synthon, 99.5%), 4‐Heptyloxybenzaldehyde (HBA, TCI Chemicals, 98%), Silicone oil (≈10 cP, Sigma–Aldrich), Silicone oil (≈100 cP, Sigma–Aldrich), Tetradecyltrimethylammonium bromide (TTAB, Sigma–Aldrich), Hexadecyltrimethylammoniumbromide (CTAB, Sigma–Aldrich), Hexadecyl‐trimethylammoniumchloride (CTAC, Sigma–Aldrich) Platinum(II) acetylacetonate (Pt(acac)2, Sigma–Aldrich, ≥99%), Iron(III) acetylacetonate (Fe(acac)3, Sigma–Aldrich, ≥99%), Oleic acid (Sigma–Aldrich, 90%), Oleylamine (Thermo Scientific Chemicals, 80–90%), Hexadecyltrimethoxysilane (Sigma‐Aldrich, ≥85%), Nile Red (Sigma–Aldrich), Polystyrene particles (Microparticles: 3 µm, PS‐R‐3.0).

### Synthesis of FePt Nanoparticles

L_10_ phase FePt nanoparticles are synthesized based on previously reported protocols by Yu, Y. et al.^[^ ^50^ ^]^ 0.2 mmol of platinum (II) acetylacetonate, 0.2 mmol of iron (III) acetylacetonate, and 0.1 mmol of cetyltrimethylammonium chloride are added in a round‐bottom flask. Afterwards, 0.5 ml Oleic acid (OA) and 10 ml of Oleylamine are added to the mixture and the flask is purged with argon gas for 1 h at room temperature. The synthesis is carried out under Argon atmosphere and reflux conditions at 340°C for 3 h. Followed by cooling the system to room temperature. FePt particles are extracted from the reaction mixture by centrifugation at 8000 rpm for 3 min and subsequent washing with hexane: ethanol (1:2 V/V) mixture. This process is repeated for five times using centrifugation. Afterward, FePt particles are washed with chloroform for 3 times and dispersed in chloroform for storage.

### Droplet Production

For the production of droplets encapsulating FePt magnetic particles, ≈20 mg of FePt nanoparticles were obtained after drying out chloroform. These particles are dispersed in 1 ml of the desired oil. 8CB, HBA, and 2 silicone oils of viscosity 10 and 100 cP were chosen. The oil particle mixture was sonicated for 2 min to ensure good dispersion. Afterward, 10 µL of the oil‐particle mixture was immediately placed in 1 wt.% TTAB solution (only in the case of HBA droplets we use 1 mM CTAB solution as the aqueous environment). Droplets of varying sizes are produced by the shake emulsification method. The produced droplets contain varying amounts of magnetic material. Therefore, a permanent magnet was used to isolate droplets with higher magnetic content. These isolated droplets are carefully pipetted out in 20 µL volumes (three to four times) and redispersed in a fresh aqueous solution of 1 wt.% TTAB (for 8CB, and silicone oils) and 1 mm CTAB (for HBA droplets). For only visualizing the chemical trail experiments, we mix Nile Red (0.6 wt.%) with 8CB before producing the droplets.

### Experimental Protocols

Surface rolling experiments were carried out in a glass chamber with 2 mm‐thick PDMS (polydimethylsiloxane) spacer. A small amount of the droplets with varying magnetic material are placed inside the chamber. The chamber was then sealed with a glass cover slip and transferred to an optical microscope (Zeiss; Axio Imager 2) equipped with a temperature chamber. Surface rolling experiments are carried out at room temperature for silicone oils. To study the rolling of nematic 8CB droplets, the experimental chamber was heated to 35°C (nematic window for 8CB: 33.5–38°C). Out‐of‐plane rotating fields were applied for surface rolling motion, using a 5‐Axis electromagnetic coil setup.

Self‐propulsion experiments are carried out in 160 µm glass chamber. For 8CB droplets, the chamber is prefilled with 20 wt.% TTAB solution and heated up to 37°C. The 8CB droplets start to demonstrate self‐propulsion from 5 wt.% TTAB. The propulsion remains steady‐state i.e. Marangoni flows with dipolar symmetry till 20 wt.% and the motion of the droplet is ballistic from 5 to 20 wt.% surfactant. Beyond 20 wt.%, the droplets begin to demonstrate jittery propulsion. In all experiments with 8CB, we used TTAB as a stabilizer for the emulsions and initiator for self‐propulsion (at 20 wt. %) as the droplets internal dynamics were in the stable regime. Except for particle adsorption experiments, where 0.08 m CTAB was used to initiate self‐propulsion as CTAB was used as a stabilizer for HDTMS modified silica particles. For HBA droplets, the experiments were carried out at room temperature and the chamber was prefilled with 10 mm CTAB solution.

### Navigation Experiments in Confined Spaces

As confined spaces, positive molds were printed using two‐photon polymerization‐based 3D microprinting (Nanoscribe Photonic Professional GT). PDMS was cast onto these molds and created the chambers. The chambers were then bonded to glass slides after oxygen plasma treatment. Afterward, the chambers were filled with 5 wt. % TTAB. Static droplets were introduced at one side of the chamber. For chemotactic experiments, a chemical gradient was created by adding a few solid TTAB crystals on the other side. The two ends were sealed using glass coverslips and introduced them into the imaging setup with a temperature pre‐heated to 37°C. Chemical gradients were visualized by mixing a small amount of Nile Red (florescent dye) with the solid TTAB. Nile Red is insoluble in water, therefore, only TTAB micelles carry it and allow for such visualization. For Marangoni assisted navigation through the chemically non‐isolating regions (isotropic conditions) and entering narrow spaces, the entire chamber was prefilled with 20 wt.% TTAB, droplets were introduced on one side and the chamber was sealed. For surface rolling assisted navigation, the chamber was prefilled with 5 wt.% TTAB, was introduced on one side and the chamber was sealed. All experiments were carried out at 37°C.

### Surface Modification of Mesoporous Silica Microparticles

A small amount of silica particles (20 µm in diameter) was dispersed in 10 mL ethanol and add 0.2 mL aqueous ammonia solution (25%) was to act as a catalyst. Followed by the introduction of 0.5 mL of Hexadecyltrimethoxysilane (HDTMS) and a vortex of the mixture for 24 h. Afterward, the particles were purified by centrifugation and washed with ethanol for five times. Finally, the particles were dried in a glass vial and dispersed them in 1 mL of 0.08 m CTAB (80 times CMC: 1 mm) solution.

### Droplet Tracking and Analysis

Experimental videos were analyzed using a custom Python script, trackpy,^[^ ^51^ ^]^ and openCV libraries. Particle speeds and trajectories were extracted from the detected particle positions in each frame.

### Simulations

Computational fluid dynamic analysis was performed using COMSOL Multiphysics 6.2 (COMSOL, Inc.). The analysis was carried out by solving the Navier‐stokes equations using the laminar flow module. A 3D simulation environment was used, with droplet defined as a sphere of radius R with specified viscosity. The oil/water interface was defined by sharp viscosity contrast. The bonding number and the capillary number for the studied droplets is <<1, therefore, the droplets were expected to remain spherical while stationary or during rolling. The droplet was studied in a semi‐infinite chamber of dimension 40R × 40R × 40R. The magnetic cluster was modeled as a rotating wall within the droplet. A finer mesh was introduced in the 3R × 3R × 3R region and within the droplet. The simulations were carried out by varying the lubrication factor for the droplets, frequency of rotation, and position of the rotating cluster for droplets of different dynamic viscosities.

## Conflict of Interest

The authors declare no conflict of interest.

## Author Contributions

M.T.A.K. conceived and designed the research idea, performed all the experiments, and wrote the manuscript. G.G. assisted in experimental procedures and helped in data analysis and interpretation. U.B. assisted with simulations and analysis of experimental results. M.S. participated in the study conception and design and supervised the research. All the authors participated in manuscript editing.

## Supporting information



Supporting Information

Supplemental Movie 1

Supplemental Movie 2

Supplemental Movie 3

Supplemental Movie 4

Supplemental Movie 5

Supplemental Movie 6

Supplemental Movie 7

Supplemental Movie 8

## Data Availability

The data that support the findings of this study are available from the corresponding author upon reasonable request.

## References

[1] R. Adkins , I. Kolvin , Z. You , S. Witthaus , M. C. Marchetti , Z. Dogic , Science 2022, 377, 768. 35951710 10.1126/science.abo5423

[2] J. Deschamps , V. Kantsler , E. Segre , V. Steinberg , Proc. Natl. Acad. Sci. U.S.A. 2009, 106, 11444. 19553213 10.1073/pnas.0902657106 PMC2710699

[3] B. A. Grzybowski , G. M. Whitesides , J. Phys. Chem. B 2002, 106, 1188.

[4] W. Wang , G. Gardi , P. Malgaretti , V. Kishore , L. Koens , D. Son , H. Gilbert , Z. Wu , P. Harwani , E. Lauga , C. Holm , M. Sitti , Sci. Adv. 2022, 8, abk0685. 10.1126/sciadv.abk0685 PMC8759740 35030013

[5] S. M. Morrow , I. Colomer , S. P. Fletcher , Nat. Commun. 2019, 10, 1011. 30824804 10.1038/s41467-019-08885-9 PMC6397266

[6] P. Khuc Trong , J. Guck , R. E. Goldstein , Phys. Rev. Lett. 2012, 109, 028104. 23030209 10.1103/PhysRevLett.109.028104

[7] R. E. Goldstein , I. Tuval , J.‐W. van de Meent , Proc. Natl. Acad. Sci. U.S.A. 2008, 105, 3663. 18310326 10.1073/pnas.0707223105 PMC2268784

[8] C. P. Brangwynne , C. R. Eckmann , D. S. Courson , A. Rybarska , C. Hoege , J. Gharakhani , F. Jülicher , A. A. Hyman , Science 2009, 324, 1729. 19460965 10.1126/science.1172046

[9] S. Birrer , S. I. Cheon , L. D. Zarzar , Curr. Opin. Colloid Interface Sci. 2022, 61, 101623.

[10] S. Michelin , Annu. Rev. Fluid Mech. 2023, 55, 77.

[11] S. Herminghaus , C. C. Maass , C. Krüger , S. Thutupalli , L. Goehring , C. Bahr , Soft Matter. 2014, 10, 7008. 24924906 10.1039/c4sm00550c

[12] I. Lagzi , S. Soh , P. J. Wesson , K. P. Browne , B. A. Grzybowski , J. Am. Chem. Soc. 2010, 132, 1198. 20063877 10.1021/ja9076793

[13] C. Jin , C. Kru‐ger , C. C. Maass , Proc. Natl. Acad. Sci. U.S.A. 2017, 114, 5089. 28465433 10.1073/pnas.1619783114 PMC5441762

[14] A. Reversat , F. Gaertner , J. Merrin , J. Stopp , S. Tasciyan , J. Aguilera , I. de Vries , R. Hauschild , M. Hons , M. Piel , A. Callan‐Jones , R. Voituriez , M. Sixt , Nature 2020, 582, 582. 32581372 10.1038/s41586-020-2283-z

[15] K. D. Young , Microbiol. Mol. Biol. Rev. 2006, 70, 660. 16959965 10.1128/MMBR.00001-06 PMC1594593

[16] D. Babu , N. Katsonis , F. Lancia , R. Plamont , A. Ryabchun , Nat. Rev. Chem. 2022, 6, 377. 37117430 10.1038/s41570-022-00392-8

[17] Metin Sitti , Mobile Microrobotics, The MIT Press, Cambridge MA, 2017.

[18] B. V. Hokmabad , A. Nishide , P. Ramesh , C. Krüger , C. C. Maass , Soft Matter 2022, 18, 2731. 35319552 10.1039/d1sm01795k

[19] M. Schmitt , H. Stark , Phys. Fluids 2016, 28, 012106.

[20] F. Lancia , T. Yamamoto , A. Ryabchun , T. Yamaguchi , M. Sano , N. Katsonis , Nat. Commun. 2019, 10, 5238. 31748502 10.1038/s41467-019-13201-6 PMC6868138

[21] C. Krüger , G. Klös , C. Bahr , C. C. Maass , Phys. Rev. Lett. 2016, 117, 048003. 27494501 10.1103/PhysRevLett.117.048003

[22] P. Guillamat , J. Ignés‐Mullol , F. Sagués , Proc. Natl. Acad. Sci. U.S.A. 2016, 113, 5498. 27140604 10.1073/pnas.1600339113 PMC4878504

[23] P. Patrício , C. R. Leal , L. F. V. Pinto , A. Boto , M. T. Cidade , Liq. Cryst. 2012, 39, 25.

[24] B. Vincenti , G. Ramos , M. L. Cordero , C. Douarche , R. Soto , E. Clement , Nat. Commun. 2019, 10, 5082. 31705050 10.1038/s41467-019-13031-6 PMC6841940

[25] G. Ramos , M. L. Cordero , R. Soto , Soft Matter 2020, 16, 1359. 31934708 10.1039/c9sm01839e

[26] R. Kree , A. Zippelius , Eur. Phys. J. E 2022, 45, 15. 35190887 10.1140/epje/s10189-022-00169-3 PMC8860840

[27] R. Kree , A. Zippelius , Eur. Phys. J. E 2021, 44, 6. 33599874 10.1140/epje/s10189-021-00018-9 PMC7892747

[28] R. Kree , L. Rückert , A. Zippelius , Phys. Rev. Fluids 2021, 6, 034201.

[29] M. Abkarian , C. Lartigue , A. Viallat , Phys. Rev. E 2001, 63, 041906. 10.1103/PhysRevE.63.041906 11308876

[30] A. J. Goldman , R. G. Cox , H. Brenner , Chem. Eng. Sci. 1967, 22, 637.

[31] A. J. Goldman , R. G. Cox , H. Brenner , Chem. Eng. Sci. 1967, 22, 653.

[32] C. Pozrikidis , J. Eng. Math 2017, 107, 111.

[33] C. K. V. S. , S. P. Thampi , Soft Matter. 2019, 15, 7605. 31475714 10.1039/c9sm01332f

[34] U. Bozuyuk , A. Aghakhani , Y. Alapan , M. Yunusa , P. Wrede , M. Sitti , Nat. Commun. 2022, 13, 6289. 36271078 10.1038/s41467-022-34023-z PMC9586970

[35] D. Disharoon , K. B. Neeves , D. W. M. Marr , Langmuir 2019, 35, 3455. 30726100 10.1021/acs.langmuir.8b04084 PMC6536127

[36] A. Chamolly , E. Lauga , S. Tottori , Soft Matter. 2020, 16, 2611. 32103230 10.1039/c9sm02250c

[37] P. Magrinya , P. Palacios‐Alonso , P. Llombart , R. Delgado‐Buscalioni , A. Alexander‐Katz , L. R. Arriaga , J. L. Aragones , Proc. Natl. Acad. Sci. U.S.A. 2025, 122, 2424236122. 10.1073/pnas.2424236122 PMC12002264 40131950

[38] M. T. A. Khan , G. Gardi , R. H. Soon , M. Zhang , M. Sitti , arXiv 2024, 10.48550/arXiv.2405.05889

[39] C. H. Meredith , P. G. Moerman , J. Groenewold , Y.‐J. Chiu , W. K. Kegel , A. van Blaaderen , L. D. Zarzar , Nat. Chem. 2020, 12, 1136. 33199888 10.1038/s41557-020-00575-0

[40] B. V. Hokmabad , J. Agudo‐Canalejo , S. Saha , R. Golestanian , C. C. Maass , Proc. Natl. Acad. Sci. U.S.A. 2022, 119, 2122269119. 10.1073/pnas.2122269119 PMC9214524 35679341

[41] S. Thutupalli , D. Geyer , R. Singh , R. Adhikari , H. A. Stone , Proc. Natl. Acad. Sci. U.S.A. 2018, 115, 5403. 29735679 10.1073/pnas.1718807115 PMC6003454

[42] S. E. Spagnolie , P. T. Underhill , Annu. Rev. Condens. Matter. Phys. 2023, 14, 381.

[43] A. Aghakhani , A. Pena‐Francesch , U. Bozuyuk , H. Cetin , P. Wrede , M. Sitti , Sci. Adv. 2022, 8, abm5126. 10.1126/sciadv.abm5126 PMC8916727 35275716

[44] B. V. Hokmabad , R. Dey , M. Jalaal , D. Mohanty , M. Almukambetova , K. A. Baldwin , D. Lohse , C. C. Maass , Phys. Rev. 2021, 11, 011043.

[45] E. Diller , J. Giltinan , M. Sitti , Int. J. Rob. Res. 2013, 32, 614.

[46] S. Floyd , E. Diller , C. Pawashe , M. Sitti , Int. J. Rob. Res. 2011, 30, 1553.

[47] R. Qu , G. Li , Biosensors (Basel) 2022, 12, 205. 35448265 10.3390/bios12040205 PMC9032088

[48] Y. Alapan , U. Bozuyuk , P. Erkoc , A. C. Karacakol , M. Sitti , Sci. Robot. 2020, 5, aba5726. 10.1126/scirobotics.aba5726 33022624

[49] R. Dey , C. M. Buness , B. V. Hokmabad , C. Jin , C. C. Maass , Nat. Commun. 2022, 13, 2952. 35618708 10.1038/s41467-022-30611-1 PMC9135748

[50] Y. Yu , L. He , J. Xu , J. Li , S. Jiang , G. Han , B. Jiang , W. Lei , W. Yang , Y. Hou , Nano. Res. 2022, 15, 446.

[51] D. B. Allan , T. Caswell , N. C. Keim , C. M. van der Weland , R. W. Verweij , Zenodo 2024, 10.5281/zenodo.12708864.

