# Supplementary material for: Surface Rolling Active Magnetic Emulsions
Source: Adv Sci (Weinh). 2025 Jun 10;12(32):e01866. doi: 10.1002/advs.202501866 (PMC12407311; doi:10.1002/advs.202501866)
Supplement: Supplementary file 1 — Supporting Information [file ADVS-12-e01866-s007.pdf]

ADVANCED SCIENCE
Open Access

Supporting Information

for *Adv. Sci.*, DOI 10.1002/advs.202501866

Surface Rolling Active Magnetic Emulsions

*Muhammad Turab Ali Khan, Gaurav Gardi, Ugur Bozuyuk and Metin Sitti**

Supporting Information

Surface Rolling Active Magnetic Emulsions

*Muhammad Turab Ali Khan, Gaurav Gardi, Ugur Bozuyuk, Metin Sitti**

Supplementary Text:
In our experiments, we particularly focused on the motion of the magnetic cluster under the application of out-of-plane rotating magnetic fields. Since the magnetic cluster is rotating inside a spherical emulsion, we cannot understand the motion by just considering one viewing angle. Therefore, we observed the motion of magnetic cluster from the top and side view to gain more insights on the behavior of magnetic cluster when subjected to rotating magnetic fields at varying frequencies. The top view gives an understanding of the x-y spatial positioning of the magnetic cluster inside the droplet, with respect to the rolling direction. The side view captures the z-x positioning of the magnetic cluster which we realized by building a setup for these experiments (Figure S1). The two viewing perspectives (Figure S2) give a complimentary understanding of the motion and behavior of the magnetic cluster at different frequencies of the rotating magnetic fields.

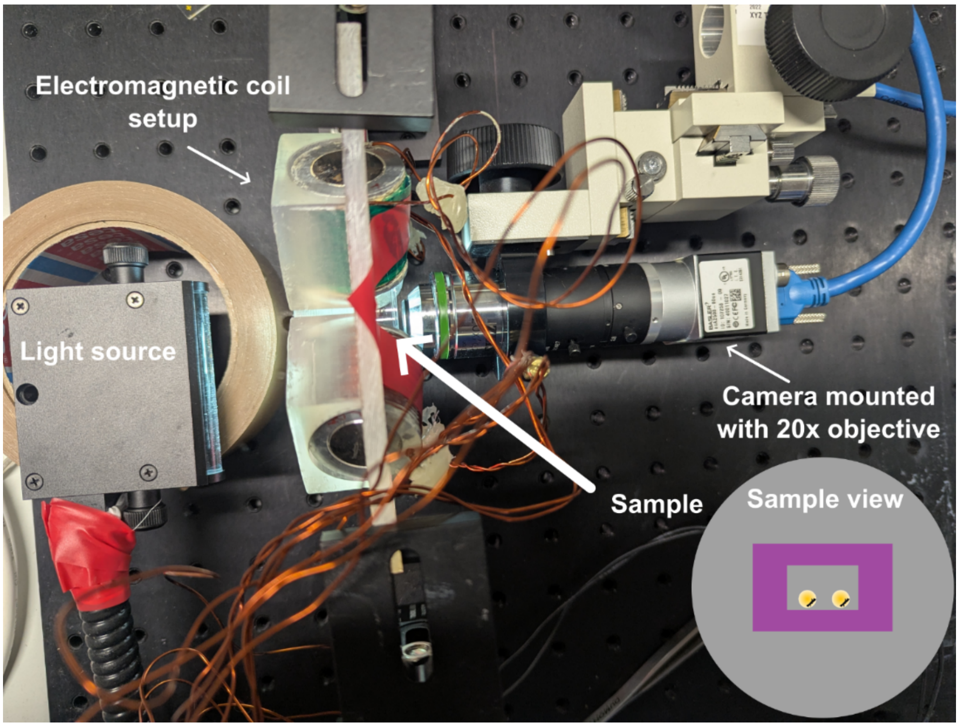

Figure S1. Custom-built experimental setup to visualize the motion of magnetic cluster in the z-x plane (side view) while performing surface rolling. The experiments are carried out at room temperature and 1 wt% TTAB. The droplets used in the experiment are nematic 5CB droplets encapsulating FePt magnetic cluster.

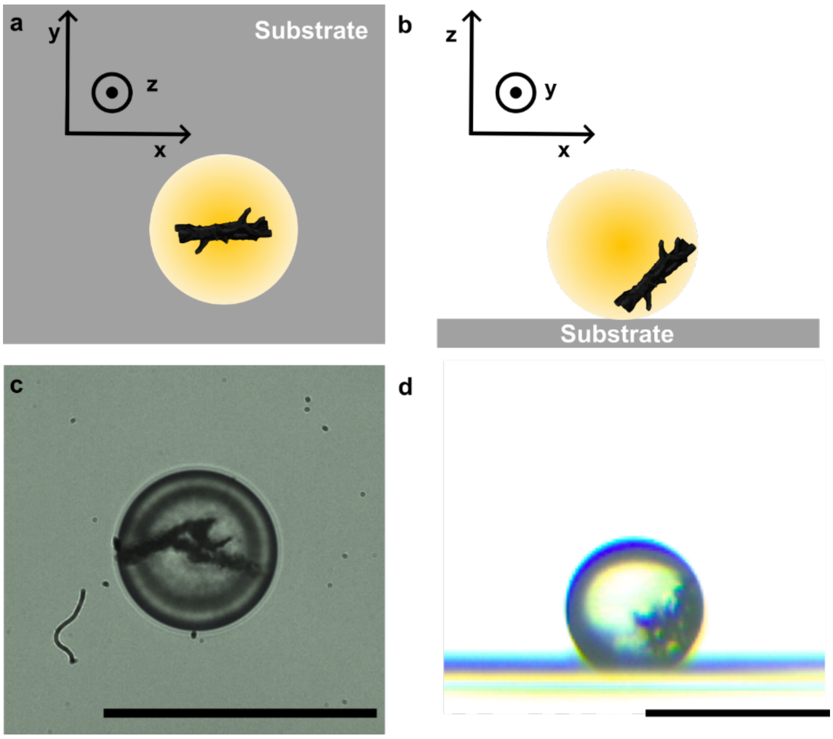

Figure S2. Two viewing perspectives of magnetic emulsions. (**a, c**) The top view of magnetic 8CB droplets on glass substrate. (**b, d**) The side view of magnetic 5CB droplets on PDMS substrate. The scale bars (c, d) represent 100 µm.

As we apply the rotating magnetic field, the magnetic cluster starts to rotate inside the droplet (Movies S1 to S3). The behavior of the rotating magnetic cluster is dependent on the frequency of the external rotating magnetic field (Movie S3 and Figure S3). At 1 Hz, we observe that the magnetic cluster is rotating at the center of the droplet (x-y plane, top view). In the z-x plane (from side view), the magnetic cluster translates and rotates close to the fluid-fluid interface. Upon increasing the frequency to 5Hz, we notice that the rotating magnetic cluster shifts to one side of the droplet (x-y plane, top view). In the z-x plane, the cluster is observed to be translating and rotating near the center of the droplet. The trajectory of the cluster inside the droplet is provided in Figure S3**.** We quantify the frequency of rotation about the center of droplet and observe that it matches with the frequency of the external magnetic field (Figure S4).

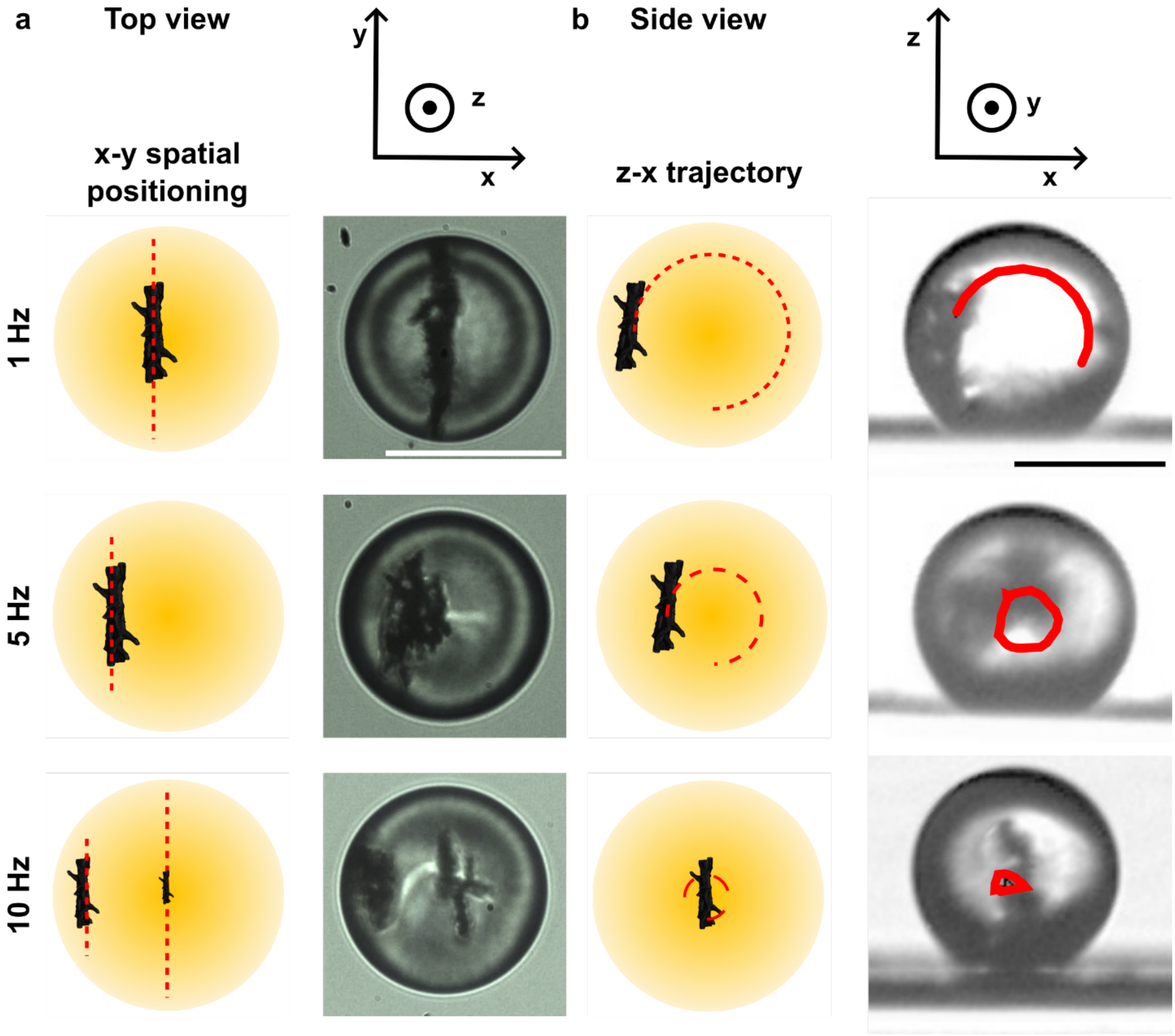

Figure S3. Motion of magnetic cluster when exposed to rotating magnetic field at 1, 5 and 10 Hz. (**a**) The x-y spatial positioning of the magnetic cluster inside nematic 8CB emulsions from top view. (**b**) The trajectory of the magnetic cluster inside nematic 5CB emulsions in the z-x plane. The scale bars represent 50 μm.

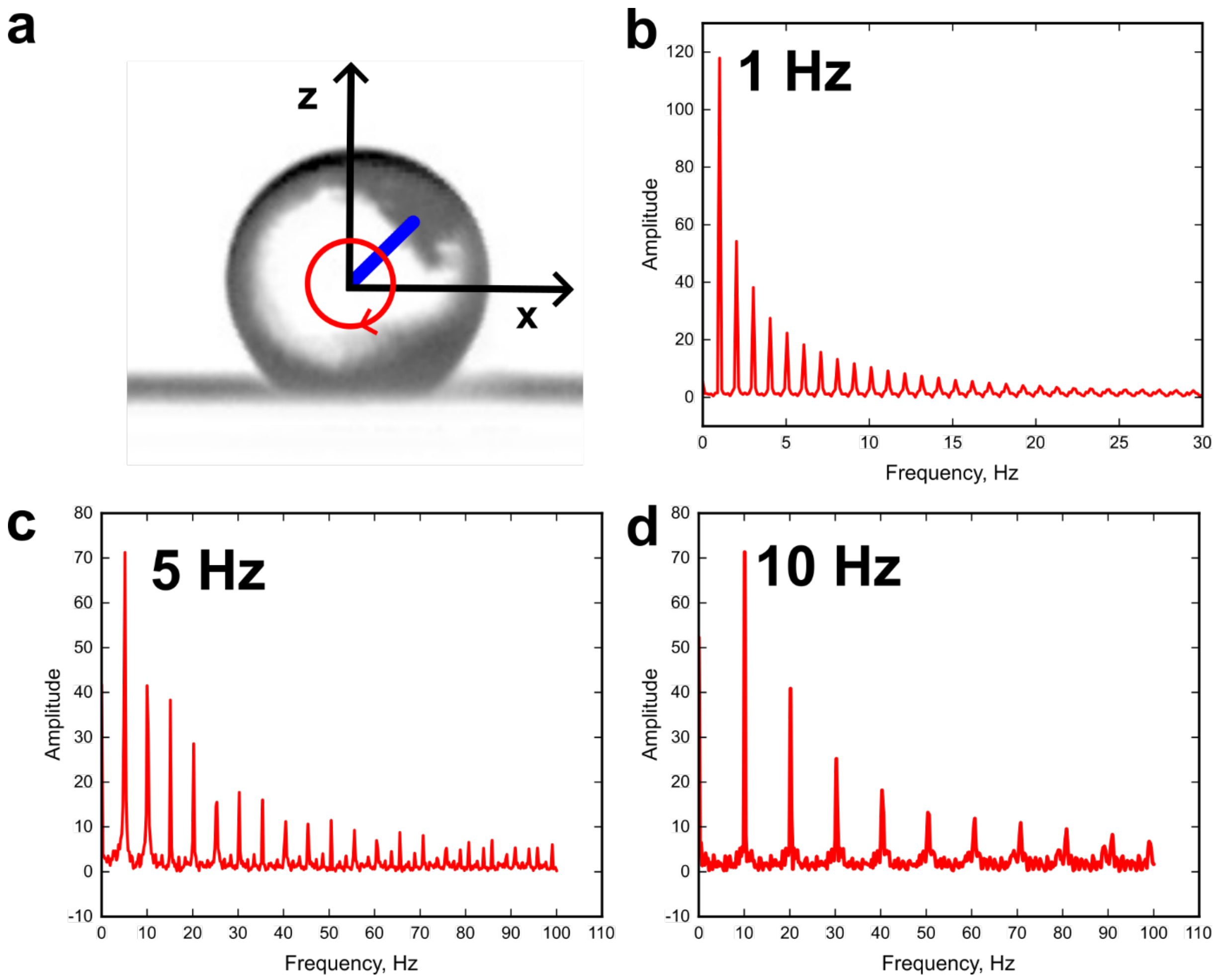

Figure S4. Rotational frequency of the magnetic cluster about the center of the droplet upon application of external magnetic field rotating at 1, 5 and 10 Hz. (a) Snapshot of a droplet rotating at 1 Hz (side view). The blue line connects the center of the droplet to the center of the cluster in the z-x plane. The red line indicates the direction of rotation of the line. (b) The Fourier transform of the angle of rotation of the blue line from (a) over time, upon exposure to rotating magnetic field at 1 Hz. (b) The Fourier transform of the angle of rotation of the blue line from (a) over time, upon exposure to rotating magnetic field at 5 Hz. (c) The Fourier transform of the angle of rotation of the blue line from (a) over time, upon exposure to rotating magnetic field at 10 Hz.

Upon further increments in the frequency (10 Hz), the magnetic cluster breaks into smaller rotating magnetic clusters (Figure S5 and Movie S3). This breaking is expected as the magnetic cluster is composed of many ferromagnetic nanoparticles held together by inter-particle magnetic dipole-dipole interactions. External magnetic field exerts a torque on the particles to align them individually along the direction of external magnetic field, while the dipole-dipole interaction tends to maintain the aggregated conformation. Upon application of rotating magnetic field at 10 Hz or beyond, the torque due to external magnetic field overcomes the particle's internal dipole-dipole torque and causes relative rotation between the magnetic particles.[3,4] This causes the cluster to break into smaller rotating clusters. It should be noted that breaking of magnetic clusters can occur in any fluid and does not explicitly depend on the confinement due to the droplet. As evident from breaking of unconfined FePt clusters at the onset of rotation in aqueous 1wt % TTAB solution (Figure S6). Furthermore, the extent of breaking of the cluster is also frequency dependent as many even smaller rotating cluster are

observed at 50 Hz (Movies S3 and Figure S5). Each of the smaller rotating cluster generates its own rotating flow profile, leading to complex trajectories of the rotating clusters and dynamic assemblies inside the emulsion.

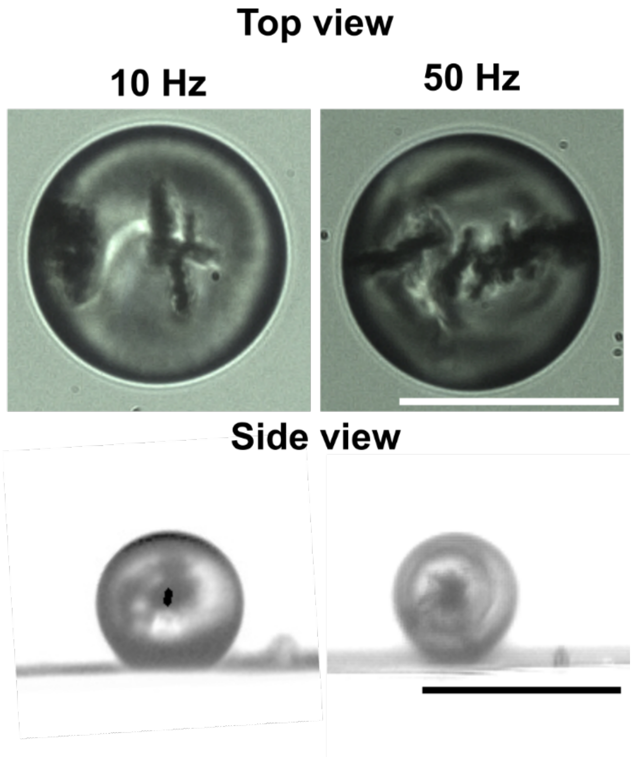

Figure S5. Snapshots (from Movie S3) showing breaking of magnetic cluster inside rolling droplets at 10 and 50 Hz. The top view of magnetic 8CB droplets rolling on glass substrate and spatial distribution of magnetic cluster in x-y plane. The side view of magnetic 5CB droplets rolling on PDMS substrate and spatial distribution of magnetic cluster in z-x plane. The scale bars represent 50 µm (Top view) and 100 µm (side view).

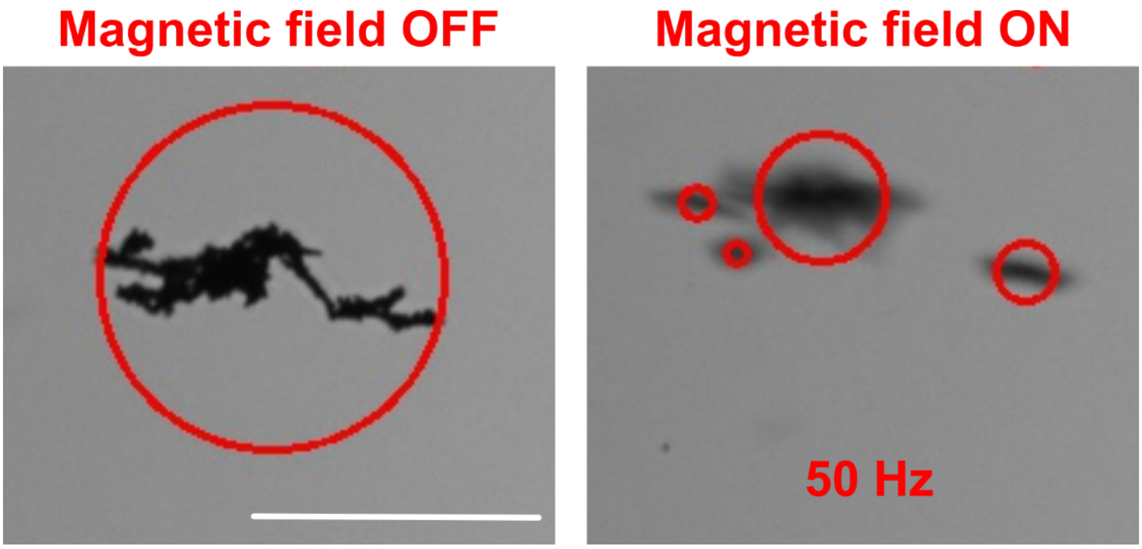

Figure S6. Breaking of FePt magnetic cluster upon exposure to out-of-plane rotating magnetic field at 50 Hz in 1 wt% TTAB solution. The scale bar represents 100 µm.

We investigate the influence of this breaking on the translation dynamics of the droplets. For a solid rotating FePt cluster (size ~ 20 µm) in 1 wt% TTAB solution, the translation speeds increase linearly with the applied magnetic field's frequency (Figure 1e). This is expected since the strength of the rotational flows increase as the frequency of rotation increases. However, the other factor that determines the strength and length scale of the rotational flows generated by a rotating rod is its size. A smaller rotating cluster generates a weaker flow compared to a bigger cluster rotating at the same frequency (Figure S7). Therefore, the breaking of the magnetic cluster affects the translation dynamics of the droplet. The reduction in effective size of the magnetic cluster and increase in rotation frequencies produce competing effects on the propulsion speed of the droplet. This leads to a non-linear trend in the translation dynamics of the droplets as observed by plateauing of the translational velocities (Figure 1d). It can be inferred that the effect of reduction in magnetic cluster's size dominates and even if the smaller magnetic clusters are rotating faster the droplet translation speed does not increase proportionally. The effect becomes more prominent for 80 µm droplets where the increase in rotational frequency beyond 30 Hz does not significantly enhance translational velocities. As the size of the droplet reduces, the rotating magnetic clusters are closer the substrate, therefore, the plateauing of velocities for 40 and 20 µm sized droplets shifts to 60 Hz (Figure S8).

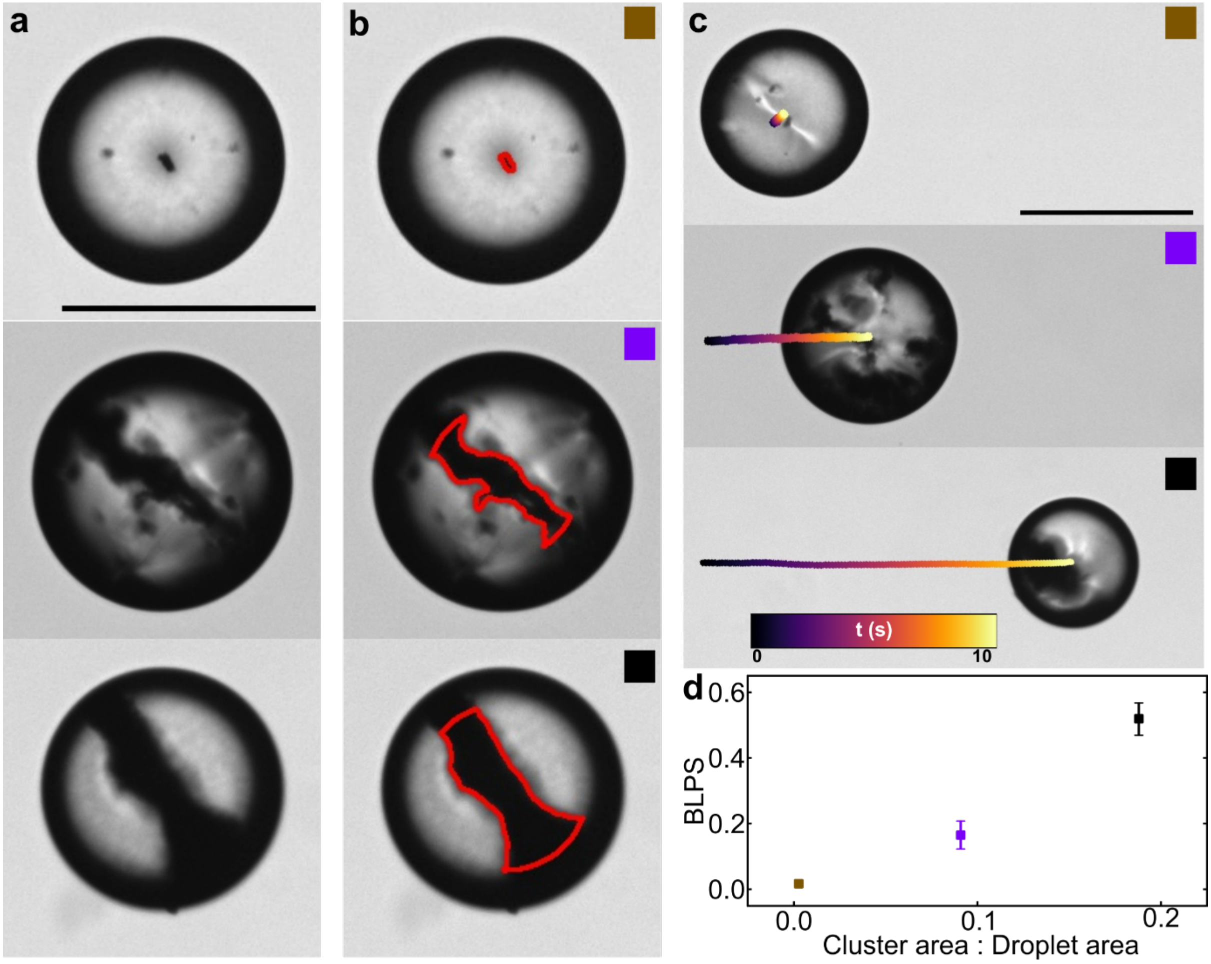

Figure S7. Effect of the size of the encapsulated magnetic cluster on the rolling dynamics of 8CB droplets (diameter = ~150 µm) in 1 wt% TTAB. (a) Snapshots of the droplets encapsulating magnetic cluster. The size of the magnetic cluster increases from top to bottom. (b) Detected area of the cluster indicated by red border of the respective images from (c) Trajectory of the droplets (first ~11 s) under the application of 10 mT, 1 Hz out-of-plane rotating magnetic fields. The trajectory is color coded with time from 0 to 11 s. (d) Speeds, normalized

as body lengths per second (BLPS), of the droplets versus the ratio of the measured cluster area to droplet area. Scale bar represents 150 µm in (a, c).

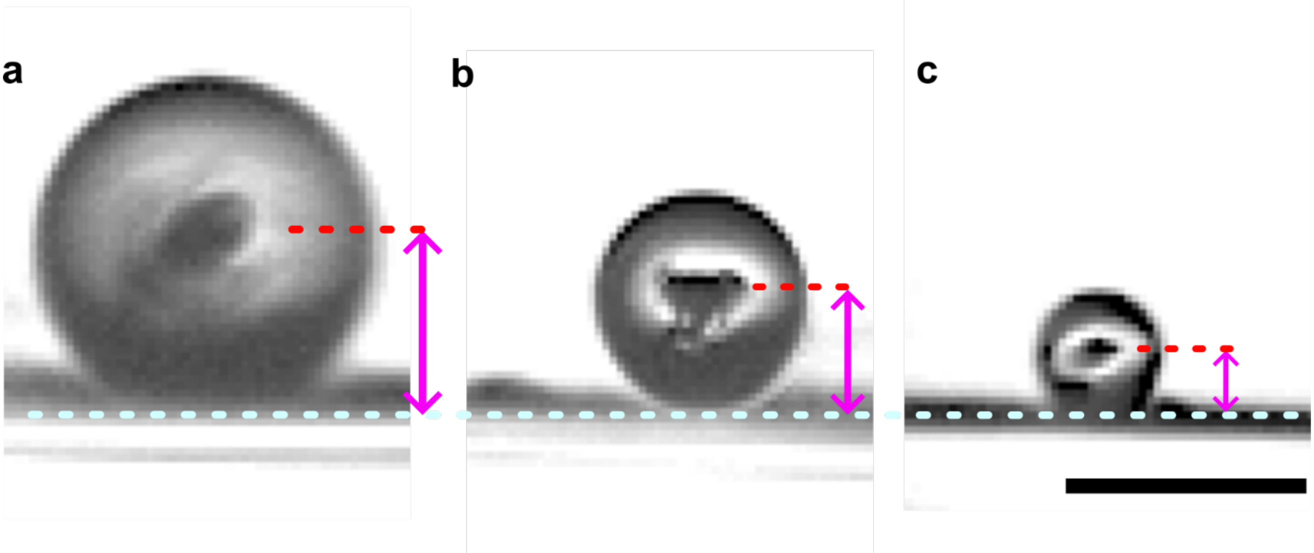

Figure S8. Snapshots of different sized droplets rolling at 50 Hz. The red line indicates the position of the rotating magnetic clusters. The double sided arrow (pink) indicates the distance from the substrate (pointed out as white). The scale bar represents 50 µm.

Shape of the experimentally studied droplets:

The shape of the static is governed by the bonding number, defined as the ratio between gravitational and surface tension effects,

$$Bo = \frac{\Delta\rho g R^2}{\sigma} = \frac{gravitational}{Surface\ tension} \quad Ca = \frac{\mu_{ext} V}{\sigma} = \frac{Shear}{Surface\ tension}$$

Assuming the surface tension to be around 35 mN m^{-1}, density = 1030 kg m^{-3} for a 80 µm 8CB system, the bonding number is 10^{-5} <<1 indicating that surface tension effects dominate and droplet's equilibrium shape should be spherical. Similarly while rolling the capillary number of the droplets is also 10^{-6} <<1 strongly indicating that surface tension effects dominate the viscous forces. Consistently, we do not observe any noticeable deformation of the droplet while stationary or rolling.

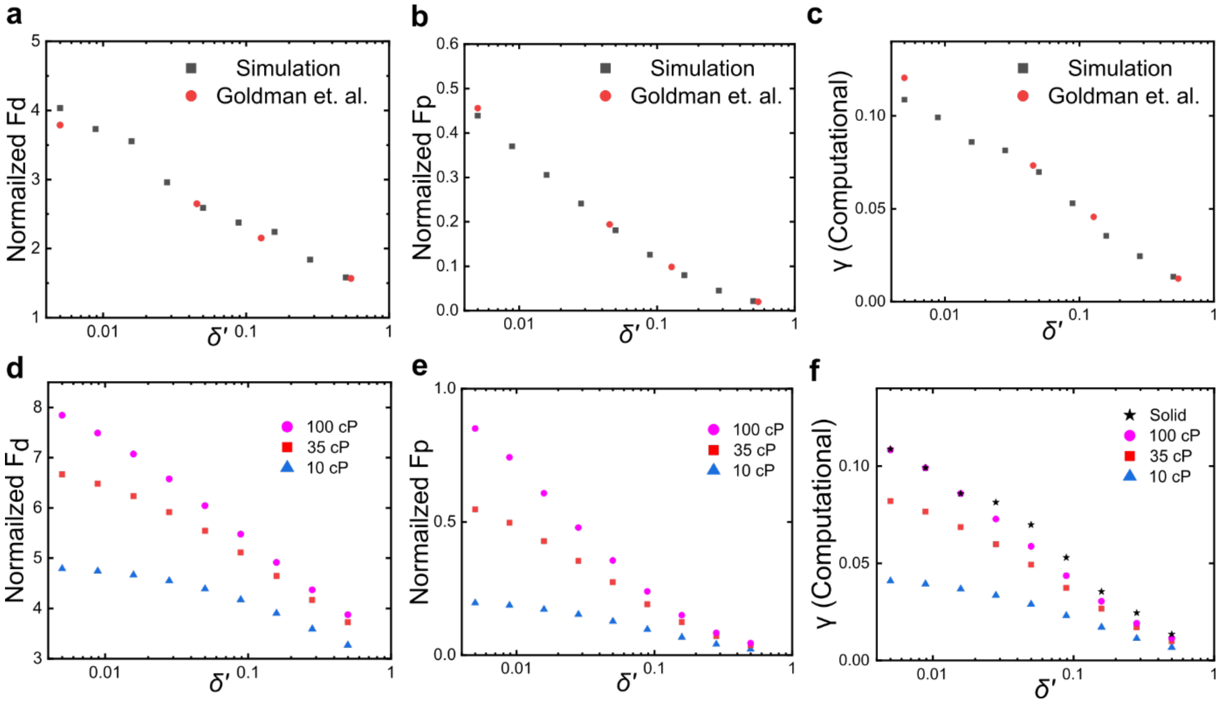

Figure S9. Comparison of the simulation results with the results reported by Goldman et. al[31]. (**a-b**) Normalized F_d and F_p for a solid spherical surface roller versus δ'. (**c**) Effective slipping coefficient $\gamma = \frac{V}{R2\pi f}$ for solid surface rollers as a function of δ'. (**d-e**) Normalized F_d and F_p for a spherical fluidic surface roller of different dynamic viscosity versus δ'. (**f**) γ for fluidic surface rollers of different dynamic viscosities in comparison with solid surface rollers as function of δ'.

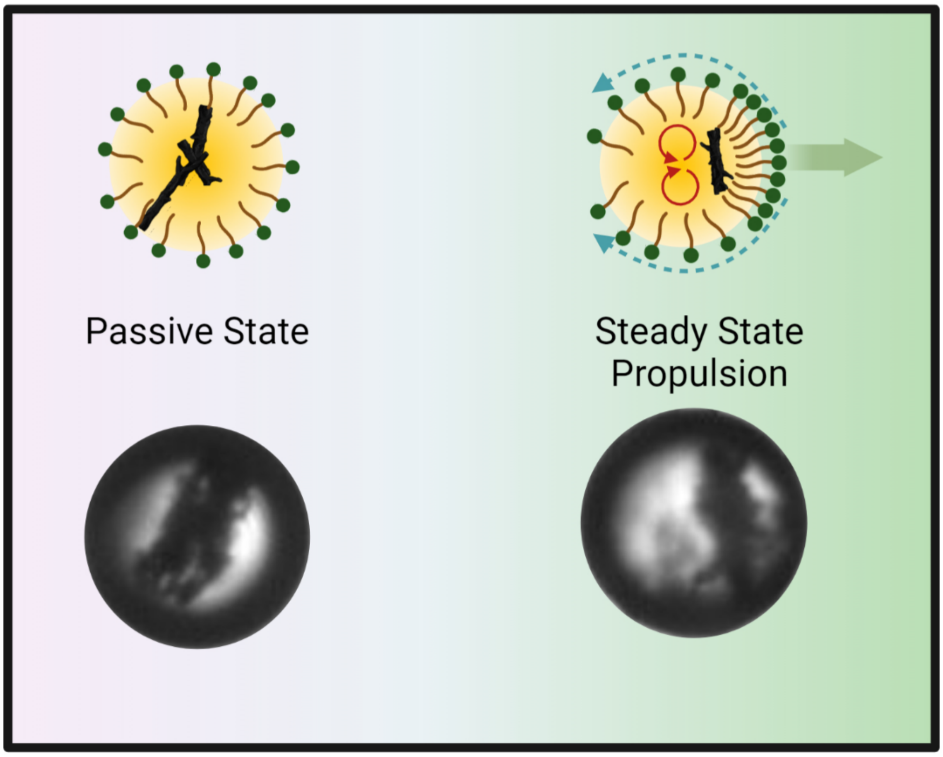

Figure S10. Positioning of the magnetic cluster with respect to droplet' state. (From Left to Right) Particles sit at the center and bottom of a static droplet in passive state. At higher surfactant concentrations, the droplet begins to perform self-propulsion due to Marangoni flows. These flows dynamically orient the magnetic cluster perpendicular to the propulsion direction.

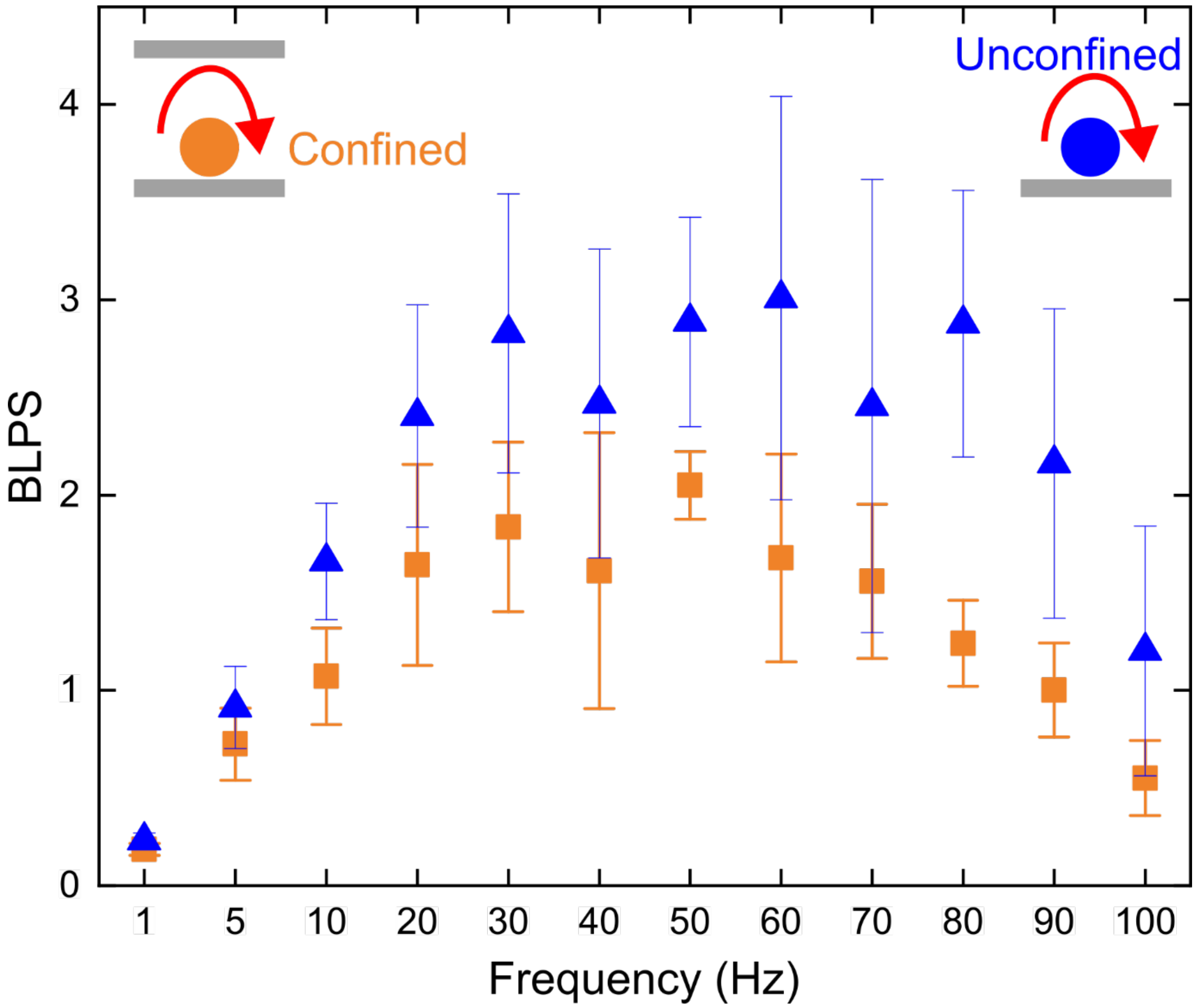

Figure S11. Comparison of the rolling speeds of ~80 µm droplets (1 wt% TTAB) in 2 body lengths confinement (chamber height = 160 µm) and unconfined droplets (chamber height = ~2 mm). Magnetic field strength is 10 mT for all cases and error bars indicate 1 standard deviation for a population of the droplets with varying cluster sizes.

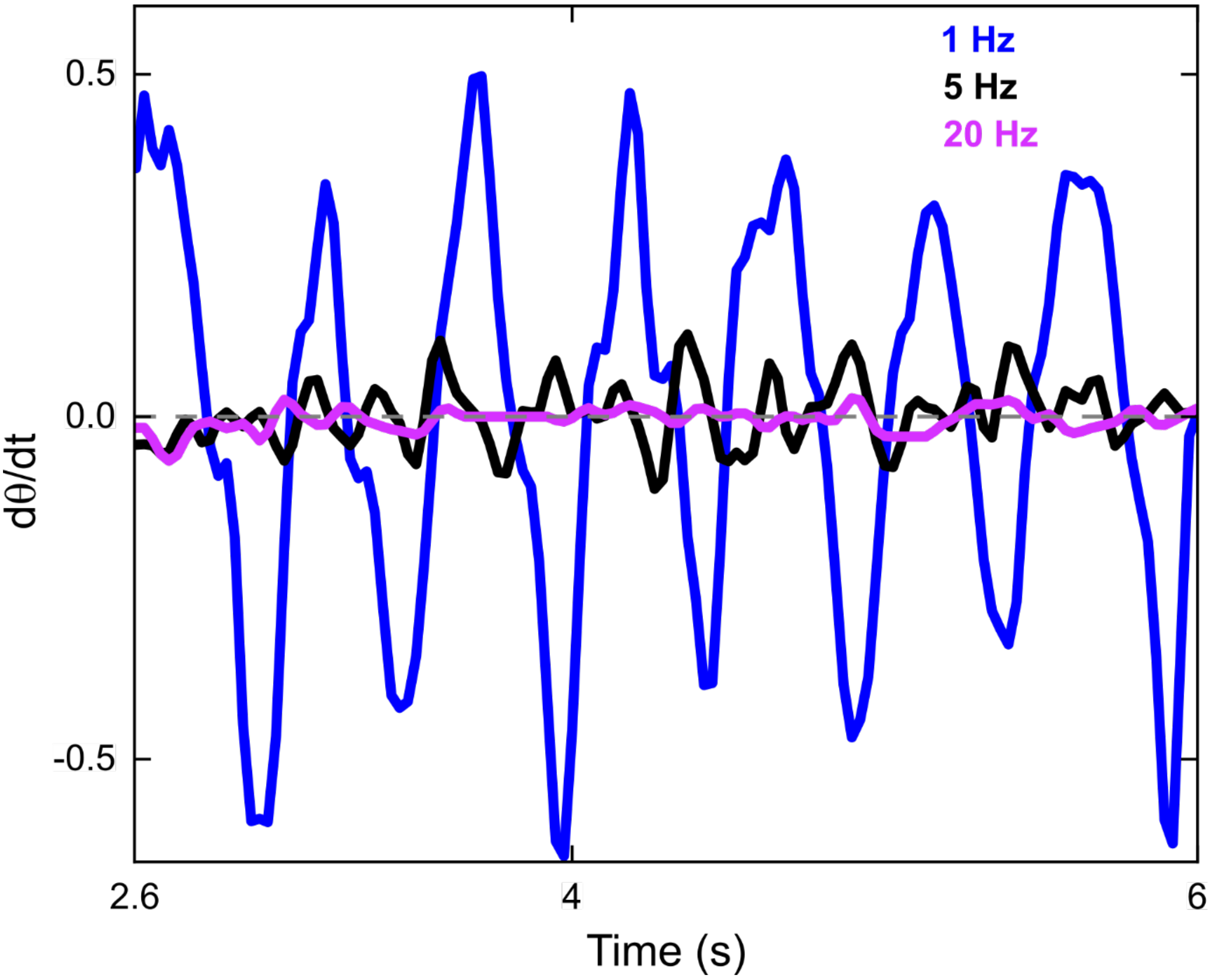

Figure S12. Comparison of the variation in the propulsion dynamics after switching to surface rolling at 20 wt% TTAB. dθ/dt, where θ represents the angle of the propulsion, is used as a measure to quantify the propulsion dynamics of the droplet at different frequencies.

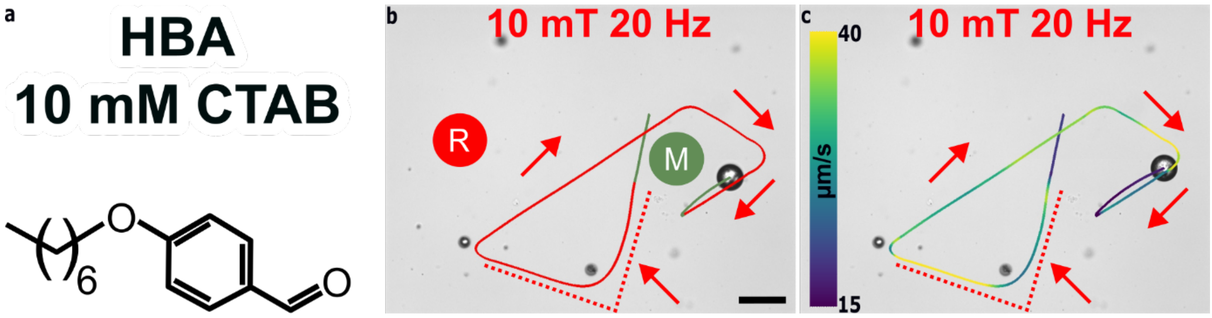

Figure S13. Demonstration of the reversible switching between self-propulsion and surface rolling for an isotropic emulsion driven by Marangoni flows. HBA (4-Heptyloxybenzaldehyde) droplets encapsulating a magnetic cluster begin to self-propel in 10 mM aqueous solutions of Cetyltrimethylammonium bromide (CTAB). (**a**) Chemical structure of HBA oil. (**b**) Trajectory of the droplet during self-propulsion (green) and surface rolling (red) at 10 mT 20 Hz. (**c**) Trajectory from (b) color coded with instantaneous speed of the droplet. Color bar indicates the instantaneous speed in µm/s. Red arrows in (b, c) indicate the direction of the plane of rotating magnetic field. Red dotted line in (b, c) marks the beginning and end of the first application of the rotating magnetic field. Scale bar in (b) indicate 100 µm.

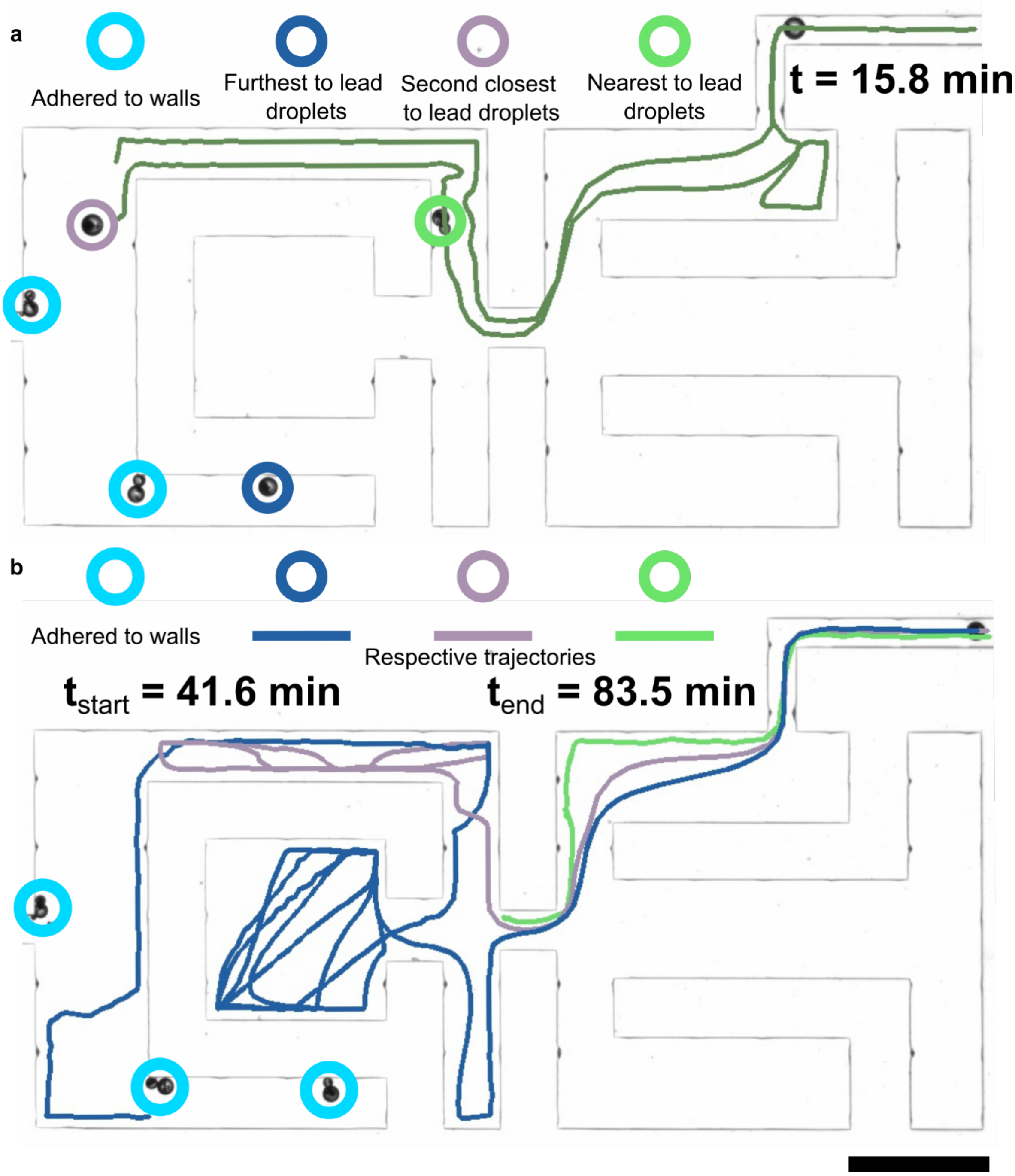

Figure S14. Trajectories of remaining droplets behind leading droplets in region with chemically isolating walls towards the chemotactic gradient. (**a**) Position of the droplets with respect the leading droplets. (**b**) Trajectories of the remaining droplets exiting the region. Scale bar represents 500 µm.

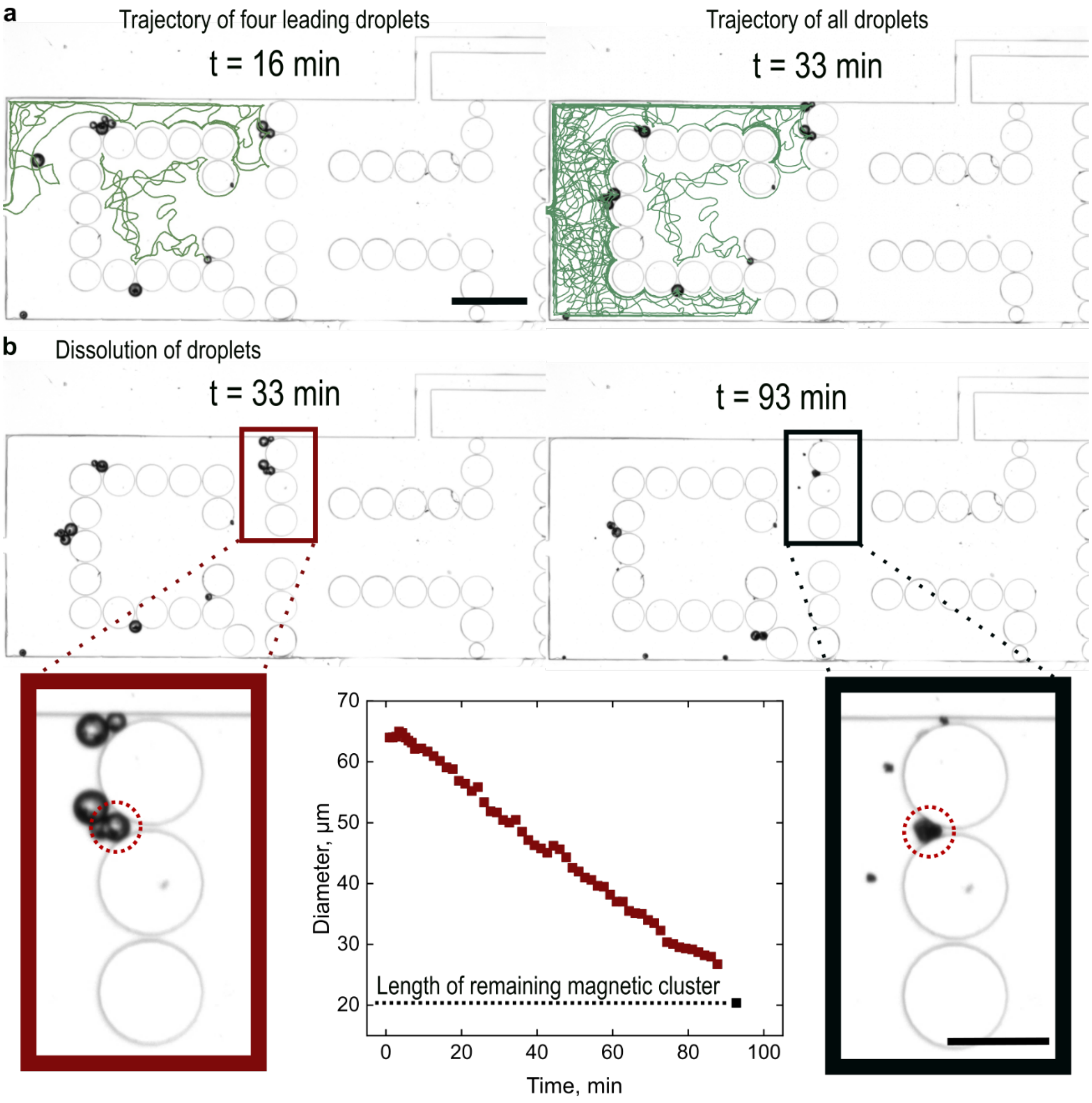

Figure S15. Trajectories and dissolution of droplets over time in chemically non-isolating regions. (**a**) Trajectories of leading 4 droplets at 16 min (left) and trajectories of all droplets in the region after 33 minutes (right). (**b**) Dissolution of leading droplets over time from time of entry in the region till 93 min. Scale bar in (**a**) represents 500 µm and in (**b**) represents 200 µm.

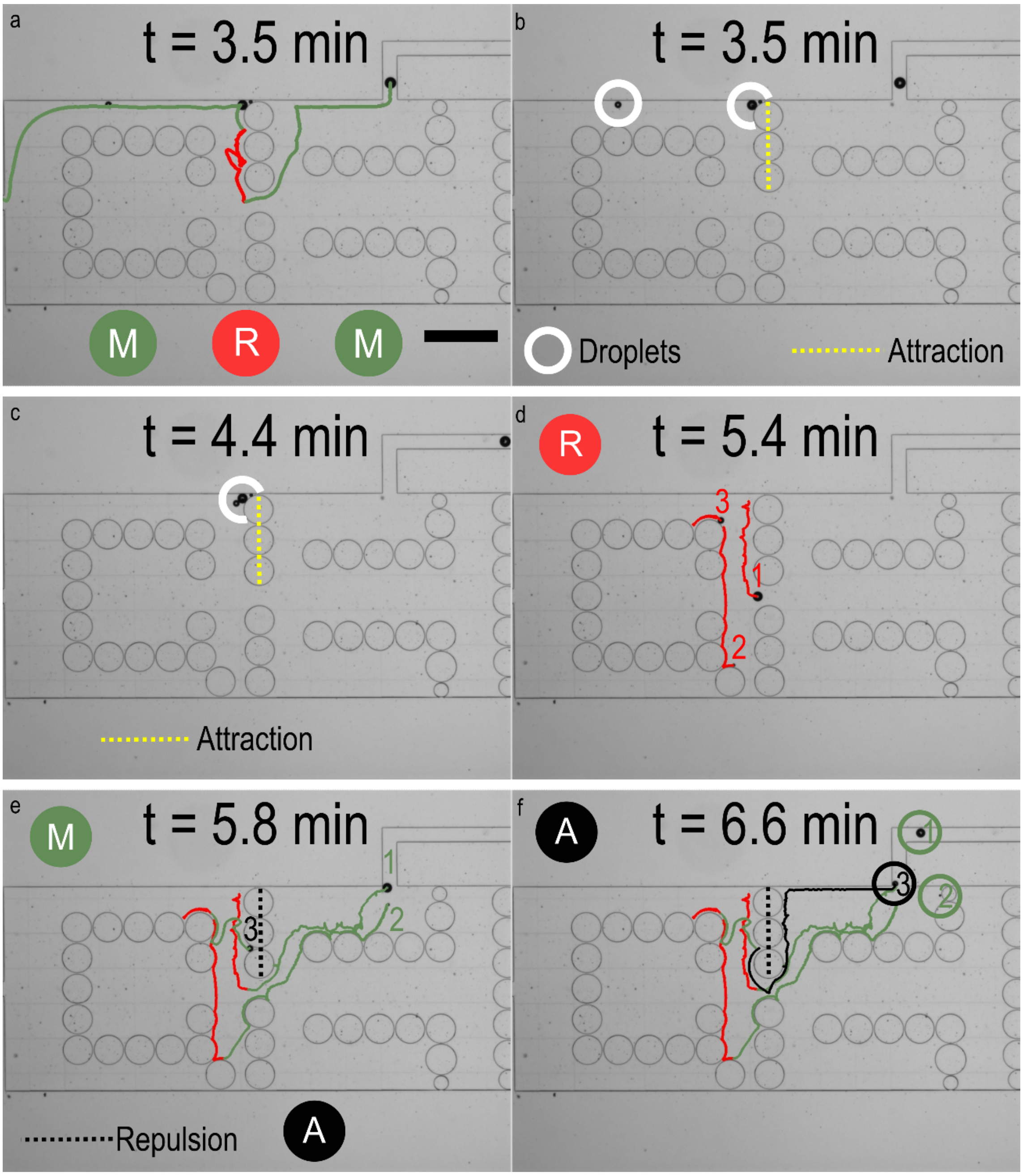

Figure S16. Auto-chemotactic interactions between the droplets give rise to reduction in attraction towards pillars in chemically non-isolating regions. (a) Trajectory of lead droplet navigating out of the region using a combination of Marangoni-assisted propulsion and surface rolling. **(b, c)** Droplets behind the lead droplet attracted towards pillars. **(d)** Switching to surface rolling to guide the remaining droplets away from pillars. **(e)** Marangoni-driven droplets guided towards outlet for droplet 1 and 2 while droplet 3 remains stuck between pillars. **(f)** Dissolution of droplet 1 and 2 causes repulsion to droplet 3 and its trajectory from resulting auto-chemotactic interactions. Scale bar represents 500 µm.

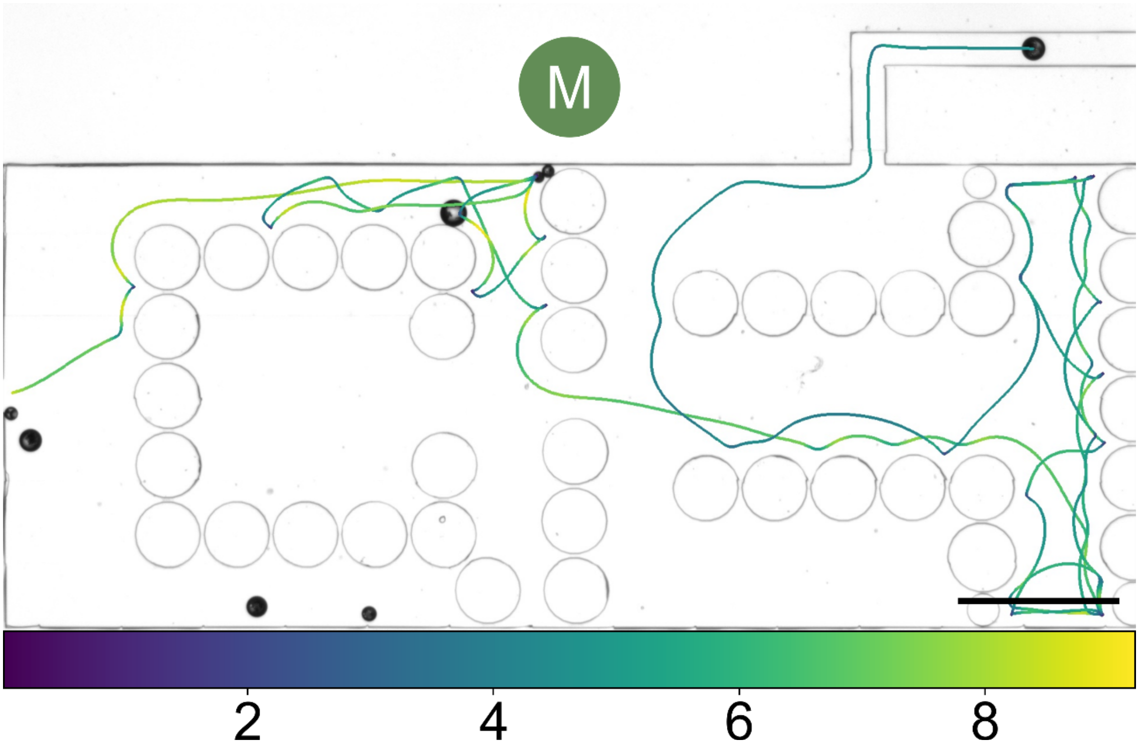

Figure S17. Trajectory of leading droplet inside chemically non-isolating region with no chemical gradient. The region is filled with 20 wt% TTAB to provide isotropic conditions. Trajectory is color coded with instantaneous speed of the self-propelling droplet, with color representing speeds in µm s^{-1}. Scale bar represents 500 µm.

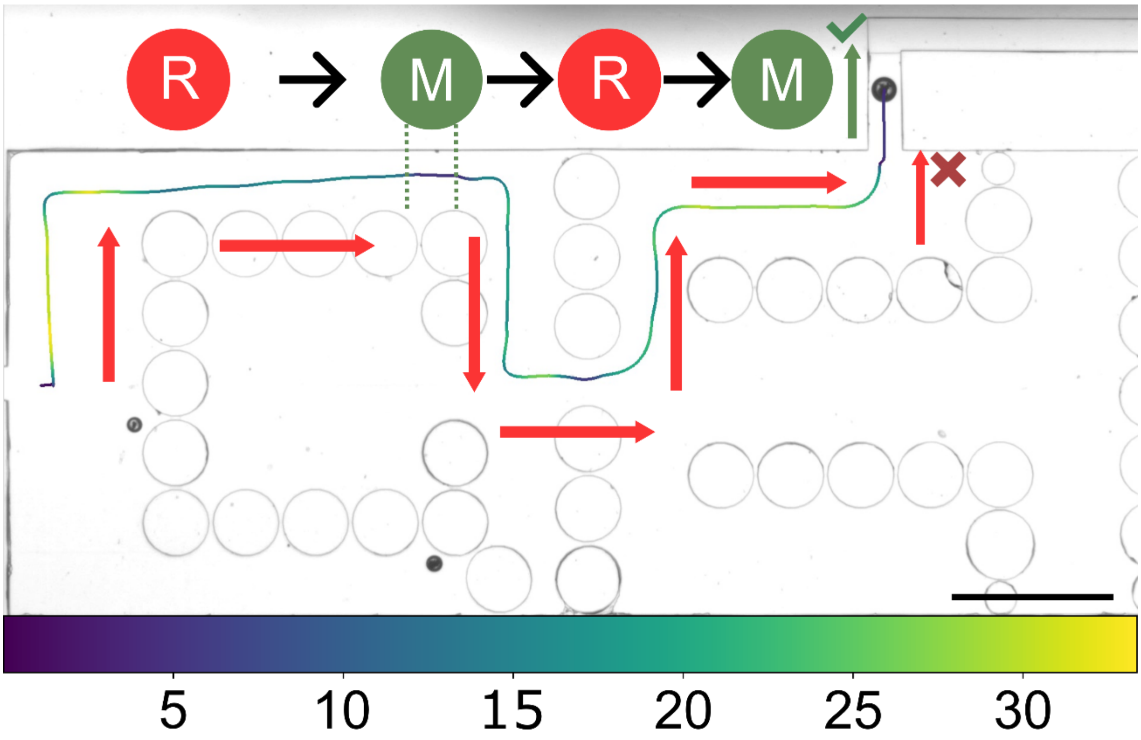

Figure S18. Surface rolling assisted navigation in chemically non-isolating regions in the presence of 5 wt % TTAB. Red arrow indicates the direction of surface rolling. Trajectories are color coded with instantaneous speed of the droplet, with color representing speeds in µm s^{-1}. Surfactant concentration of 5 wt % allows switching between surface rolling and active motion to enable entry inside narrow channel. Scale bar represents 500 µm.

Movie captions 1 to 8

Movie S1: Surface rolling droplet under out-of-plane rotating fields.
Under out-of-plane rotating fields, the magnetic cluster rotates inside a static droplet creating rotational flows within the droplet and allow for surface rolling. At 10 Hz, continuous rotational flows arise within the droplets, causing the droplets to roll on nearby boundaries. Ceasing magnetic fields, leads to restoration of the equilibrium ordering in a passive (static) droplet.

Movie S2: Surface rolling droplet (side view) at different frequencies of out-of-plane rotating fields.
A comparative video showing the motion of surface rolling droplets and the encapsulated magnetic cluster (from the side) with respect to the frequency of the external rotating magnetic field.

Movie S3: Motion and behavior of the magnetic cluster at different frequencies of out-of-plane rotating fields.
Understanding the motion of the magnetic cluster in droplet's frame of reference at 1 and 5 Hz from two viewing perspectives. The dynamic breaking and motion of the magnetic cluster at higher rotational frequencies (10 and 50 Hz).

Movie S4: Locomotion switch between self-propulsion and surface rolling using out-of-plane rotating fields.
A self-propelling droplet can switch between swimming and surface rolling under out-of-plane rotating fields. Rotational flows help overcome the Marangoni flows and enable higher propulsion speeds and extent of control. Locomotion switch to surface rolling also enables manipulation and patterning of the solubilized droplet trail.

Movie S5: Chemotactic navigation in confined spaces.
We investigate navigation ability of droplets along pre-patterned chemical gradients in chemically isolating and non-isolating regions. Droplets are able to exit in chemically isolating regions, however, navigation out of chemically non-isolating regions requires external control to extract droplets from concentration gradient traps.

Movie S6: Entering narrow channels.
We demonstrate Marangoni flow-driven droplet can autonomously enter narrow channels however, a rolling droplet cannot enter narrow channels due to hydrodynamic flows associated with rolling droplet. Switching to Marangoni propulsion leads to effortless entry into narrow channels.

Movie S7: Flow assisted carving trajectory, particle transport, patterning and release.
Hydrodynamic flow fields vary with respect to locomotion mode. Surface rolling can be used to trap cargo, carve trajectories and go against chemotactic gradients, while switching to self-propulsion, enables spontaneous release of cargo and movement along the chemotactic gradient.

Movie S8: Transporting and releasing adsorbed particles.
Fluidic nature of droplets enables cargo transport by adsorption on their body. A droplet is magnetically steered towards potential cargo and cargos are dragged along the guided path. Adsorbed particles are released by switching to surface rolling.
